# Retrovirus-based pseudotyped virus neutralisation assays overestimate neutralising activity in sera from participants receiving integrase inhibitors

**DOI:** 10.1038/s41598-025-11362-7

**Published:** 2025-08-05

**Authors:** Mhairi J. McCormack, Patawee Asamaphan, Ellen C. Hughes, Louis Banda, Stephen Kasenda, Chris Davis, Agnieszka M. Szemiel, Amelia Crampin, Abena S. Amoah, Emma C. Thomson, Antonia Ho, Brian J. Willett

**Affiliations:** 1 Medical Research Council, University of Glasgow Centre for Virus Research, Glasgow, UK; 2 Department of Livestock and One Health, Institute of infection, veterinary and ecological sciences, University of Liverpool, Liverpool, UK; 3 Malawi Epidemiology and Intervention Research Unit (MEIRU), Lilongwe, Malawi; 4 London School of Hygiene and Tropical Medicine, London, UK; 5 School of Health and Wellbeing, University of Glasgow, Glasgow, UK; 6 Leiden University Medical Center, Leiden, Netherlands

**Keywords:** Pseudovirus assay, Interference, Integrase inhibitors, HIV, Biological techniques, Immunology, Virology

## Abstract

Retroviral pseudotype-based virus neutralisation assays are widely used to estimate functional immunity, but may be unsuitable for testing human immunodeficiency virus (HIV)-infected individuals receiving integrase inhibitor treatment. We evaluated these assays for measuring severe acute respiratory syndrome coronavirus 2 (SARS-CoV-2) and hepatitis C virus (HCV) neutralisation in people living with HIV. SARS-CoV-2 neutralisation was assessed using HIV-based SARS-CoV-2 pseudoviruses in sera from a longitudinal Malawian cohort (*n* = 1,876), detecting neutralisation across timepoints in 10.5–54.5% of HIV-uninfected vs. 85.5–93.9% of HIV-infected participants (*n* = 96). HIV-infected sera were re-tested using vesicular stomatitis virus (VSV)-based SARS-CoV-2 pseudoviruses, estimating seroprevalence at 5.6–65.2%, suggesting HIV-based assays overestimate neutralisation. HIV-based VSV-glycoprotein(G) pseudoviruses confirmed non-specific inhibition in 75.0-87.9% of HIV-infected participants. HCV neutralisation was assessed in UK-based HCV patients (*n* = 100, *n* = 90 HIV-infected) using murine leukaemia virus (MLV)-based HCV pseudoviruses. Non-HCV-specific inhibition was detected in integrase inhibitor recipients. Median neutralisation of MLV(HCV) pseudoviruses was higher in integrase inhibitor recipients (71.8% vs. 21.3%). Testing HIV(SARS-CoV-2) and MLV(HCV) pseudoviruses against antiretroviral drugs showed interference by integrase inhibitors (specifically, dolutegravir). Isolating IgG from serum removed the interference by residual drugs. Retrovirus-based assays are therefore unsuitable for testing individuals receiving integrase inhibitors. Protective immunity to viruses has likely been over-reported in HIV prevalent populations.

**Supplementary Information:**

The online version contains supplementary material available at 10.1038/s41598-025-11362-7.

## Introduction

Virus neutralisation assays measure antibodies that inhibit infection. The presence of these neutralising antibodies is a strong indicator of protection against future symptomatic infections^1–3^. Neutralisation assays are instrumental in measuring population level protective immunity; understanding cross neutralisation; evaluating vaccine efficacy; and identifying groups with weak neutralising antibody responses at risk of severe disease^4–12^. Pseudotyped virus neutralisation assays (PVNAs) offer a practical alternative to live virus neutralisation assays as they are replication-defective and can, therefore, be performed in biosafety level (BSL)-2 laboratories, while live virus work requires BSL-3 laboratories^13,14^. Further, live virus neutralisation assays are more labour-intensive than PVNAs, making them impractical for high-throughput screening^15^.

Retrovirus-based assays in which viral glycoproteins are expressed on either human immunodeficiency virus (HIV) or murine leukaemia virus (MLV) particles have been widely used, notably in severe acute respiratory syndrome coronavirus 2 (SARS-CoV-2) and hepatitis C virus (HCV) research^16^. A number of South African studies that used retrovirus-based assays excluded participants living with HIV^17–19^, as a previous study indicated that certain antiretroviral therapy (ART) drugs (integrase or reverse transcriptase inhibitors) might interfere with HIV(SARS-CoV-2) PVNAs, possibly by preventing reporter gene expression from the HIV vector^20^. Another study reported that integrase inhibitors specifically interfere with HIV(SARS-CoV-2) systems^21^. These studies did not examine interference in other retrovirus-based PVNA systems, as they focused only on HIV(SARS-CoV-2) assays. Additionally, they lacked comprehensive mechanistic analysis. One study documented that antibodies against HIV might cross-neutralise SARS-CoV-2, without addressing potential interference with their HIV-based assay^22^.

To clarify the issues underlying the use of retrovirus-based neutralisation assays with samples from people living with HIV, we examined two HIV-prevalent cohorts. Firstly, a longitudinal Malawi-based cohort, where SARS-CoV-2 neutralisation was assessed using HIV(SARS-CoV-2) pseudotypes^23^. Secondly, a UK-based cohort of HCV-infected patients, where HCV neutralisation was assessed using MLV(HCV) pseudotypes. We compared findings from the retrovirus-based PVNAs with results from vesicular stomatitis virus (VSV)-based PVNAs and investigated whether interference was dependent on the SARS-CoV-2 and HCV surface glycoproteins by comparing with pseudotypes bearing the VSV-glycoprotein(G). We hypothesised that antiretrovirals might cause non-specific interference in retrovirus-based neutralisation assays, and investigated if ART use (and for the UK cohort, integrase inhibitor use) affected assay results.

## Results

### High SARS-CoV-2 neutralisation using the HIV(SARS-CoV-2) pseudovirus system in HIV-infected participants

**Fig. 1:**
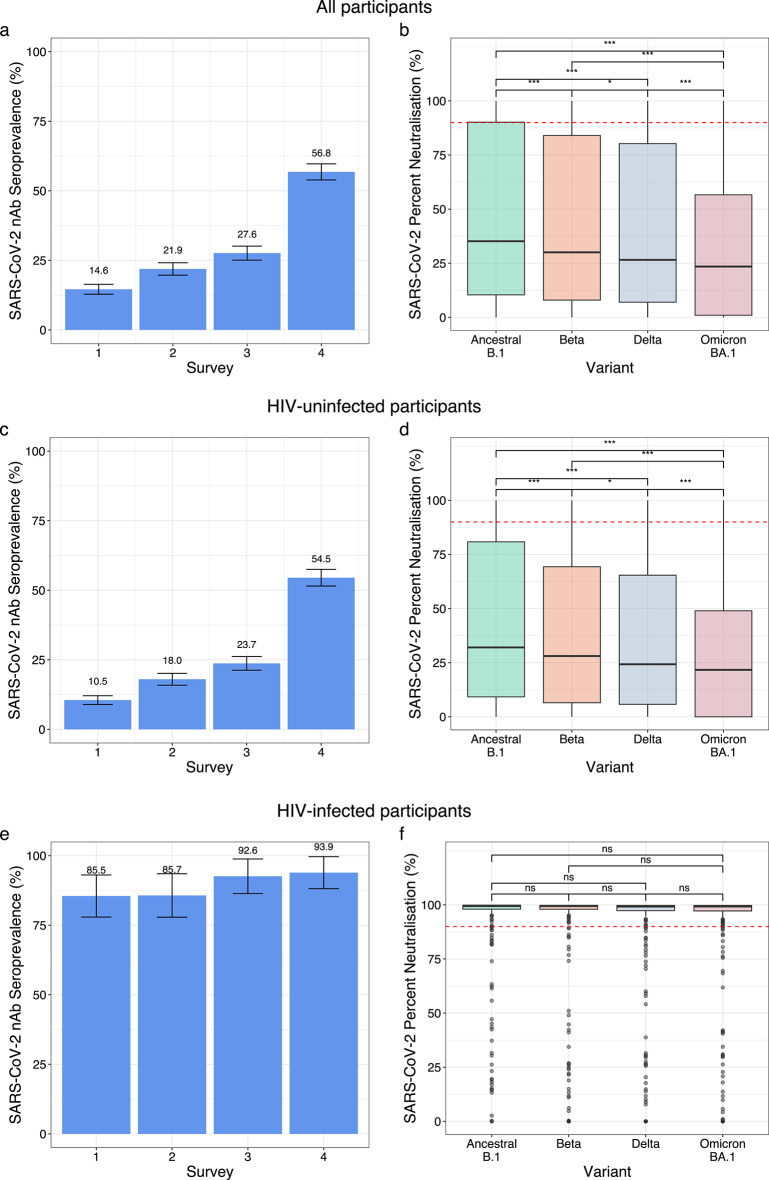
SARS-CoV-2 neutralising activity in all study participants from the Malawi cohort. Activity was assessed using the HIV(SARS-CoV-2) PVNA, and stratified by HIV status. (**a**) Estimated seroprevalence of neutralising antibodies (nAb) across four surveys for all participants (*n* = 1,876). Error bars represent 95% confidence intervals, with percentage values shown. (**b**) Percent neutralisation against Ancestral B.1, and the Beta, Delta, and Omicron (BA.1) variants across the entire study period, for all participants (*n* = 1,876). Box plots display the median and interquartile range (IQR). Red dashed line shows the 90% cut-off. Statistical significance was determined using the Wilcoxon rank-sum test. (**c**) Seroprevalence of neutralising antibodies (nAb) across four surveys for HIV-uninfected participants (*n* = 1,780). Error bars represent 95% confidence intervals, with percentage values shown. (**d**) Percent neutralisation against Ancestral B.1, and the Beta, Delta, and Omicron BA.1 variants across the entire study period in HIV-uninfected participants (*n* = 1,780). Box plots display the median and interquartile range (IQR). Red dashed line shows the 90% cut-off. Statistical significance was determined using the Wilcoxon rank-sum test. (**e**) Seroprevalence of neutralising antibodies (nAb) across four surveys for HIV-infected participants (*n* = 96). Error bars represent 95% confidence intervals, with percentage values shown. (**f**) Percent neutralisation against Ancestral B.1, Beta, Delta, and Omicron (BA.1) variants across the entire study period in HIV-infected participants (*n* = 96). Box plots display the median and interquartile range (IQR). Red dashed line shows the 90% cut-off. Statistical significance was determined using the Wilcoxon rank-sum test: ns - not significant, *p < 0.05, **p < 0.01, *** *p* < 0.001.

The Malawi cohort comprised of *n* = 1,876 participants, sera for which were collected longitudinally and tested for neutralising antibodies against SARS-CoV-2 variants. The variants tested against were dependent on those that had circulated in Malawi prior to sample collection (Supplementary Fig. 1). The prevalence of SARS-CoV-2 neutralising antibodies in this cohort, measured using the HIV(SARS-CoV-2) PVNA, increased from 14.6% (CI 12.8–16.4%) at Survey 1 to 56.8% (CI 54.0-59.7%) by Survey 4 (Fig. 1a), due to increasing SARS-CoV-2 infections in the population (Supplementary Fig. 1). Median percent neutralisation (neutralising activity estimate) was consistently highest against the Ancestral B.1 virus (35.2%, IQR 10.4–90.2%) and lowest against Omicron BA.1 (23.5%, IQR 1.0-56.6%), with significant differences between all variants (Ancestral B.1 vs. Beta, *p* = 4.6 × 10^− 6^; Ancestral B.1 vs. Delta, *p* = 1.5 × 10^− 13^; Ancestral B.1 vs. Omicron BA.1, *p* = 3.8 × 10^− 55^; Beta vs. Delta, *p* = 0.034; Beta vs. Omicron BA.1, *p* = 2.4 × 10^− 26^; Delta vs. Omicron BA.1, *p* = 1.4 × 10^− 15^, Wilcoxon test) (Fig. 1b).

In HIV-uninfected participants (94.9% of the total cohort), estimated seroprevalence ranged from 10.5% (CI 8.9–12.1%) to 54.5% (CI 51.5–57.5%) (Fig. 1c), comparable to the trend with all participants (Fig. 1a), though for Survey 1 estimated seroprevalence was significantly lower in HIV-uninfected participants (*p* = 0.0038, Chi-sq. test). Median percent neutralisation for all variants was significantly lower in HIV-uninfected participants (Ancestral B.1, *p* = 0.0019; Beta, *p* = 0.0012; Delta, *p* = 0.0012; Omicron BA.1, *p* = 3.7 × 10^− 4^, Wilcoxon) (Fig. 1d) compared with all participants (Fig. 1b) (Supplementary Table 1). The subgroup of HIV-infected participants had consistently higher estimated seroprevalence (85.5% (CI 78.0-93.1%) to 93.9% (CI 88.2–99.7%)) (Fig. 1e) than HIV-uninfected participants (Survey 1, *p* = 3.0 × 10^− 77^; Survey 2, *p* = 1.1 × 10^− 42^; Survey 3, *p* = 9.6 × 10^− 34^; Survey 4, *p* = 3.1 × 10^− 9^, Chi-sq.) (Fig. 1c). Percent neutralisation in HIV-infected participants exceeded 90% for all variants, significantly higher than in HIV-uninfected participants (Ancestral B.1, *p* = 9.2 × 10^− 93^; Beta, *p* = 5.6 × 10^− 97^; Delta, *p* = 3.9 × 10^− 99^; Omicron BA.1, *p* = 6.8 × 10^− 116^, Wilcoxon) (Fig. 1d and f).

### Interference with the HIV-based pseudovirus system was detected

**Fig. 2:**
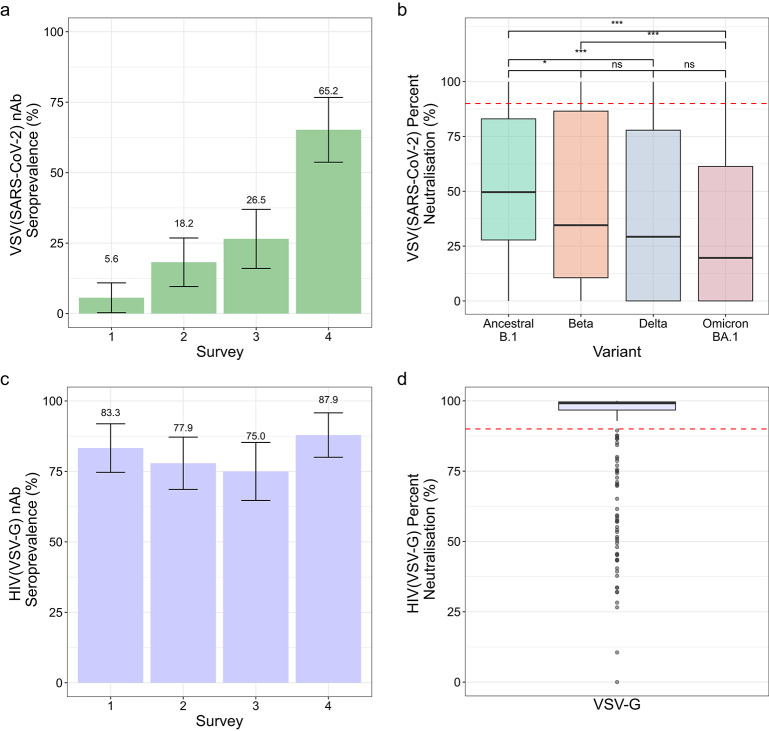
Neutralising activity in sera from HIV-infected participants (*n* = 96). Activity was assessed using the VSV(SARS-CoV-2) and HIV(VSV-G)-based assays. (**a**) Neutralising antibody (nAb) seroprevalence across the four surveys was measured using the VSV(SARS-CoV-2) PVNA. Error bars represent 95% confidence intervals, with percentage values shown. (**b**) Percent neutralisation against Ancestral B.1, and the Beta, Delta and Omicron (BA.1) variants across the entire study period, using VSV-based pseudotypes. Box plots display the median and interquartile range (IQR). Red dashed line shows the 90% cut-off. Statistical significance was determined using the Wilcoxon rank-sum test: ns - not significant, *p < 0.05, **p < 0.01, *** *p* < 0.001. (**c**) Neutralising antibody (nAb) seroprevalence across the four surveys was assessed using HIV(VSV-G) pseudoviruses to determine if non-SARS-CoV-2 S-related inhibitory activity was occurring. Error bars represent 95% confidence intervals, with percentage values shown. (**d**) Percent neutralisation against HIV(VSV-G) across the entire study period. Box plot displays the median and interquartile range (IQR). Red dashed line shows the 90% cut-off.

Given the unexpectedly high neutralising activity in the HIV-infected participants of the Malawi cohort (measured using the HIV(SARS-CoV-2) PVNA), sera from these individuals were re-tested using a VSV(SARS-CoV-2) PVNA to identify if SARS-CoV-2 neutralising antibody seroprevalence was overestimated. Estimated seroprevalence in HIV-infected participants was significantly lower with the VSV(SARS-CoV-2) PVNA compared with the HIV(SARS-CoV-2) PVNA at all surveys, ranging from 5.6% (CI 0.3–10.8%) at Survey 1 to 65.2% (CI 53.7–76.6%) at Survey 4 (Survey 1, *p* = 5.6 × 10^− 22^; Survey 2, *p* = 7.6 × 10^− 16^; Survey 3, *p* = 6.0 × 10^− 14^; Survey 4, *p* = 0.00040, Chi-sq.) (Fig. 2a). These VSV(SARS-CoV-2) assay based seroprevalence estimates are more consistent with the seroprevalence estimates in HIV-uninfected individuals using the HIV(SARS-CoV-2) assay (Fig. 1c). Therefore, the HIV(SARS-CoV-2) PVNA system likely overestimated seroprevalence in HIV-infected participants. Additionally, within the HIV-infected participants, percent neutralisation was significantly lower using the VSV(SARS-CoV-2) PVNA (Fig. 2b) compared with the HIV(SARS-CoV-2) PVNA for all variants (Ancestral B.1, *p* = 1.0 × 10^− 47^; Beta, *p* = 3.3 × 10^− 48^; Delta, *p* = 4.4 × 10^− 47^; Omicron BA.1, *p* = 2.1 × 10^− 46^, Wilcoxon) (Fig. 1f).

We investigated whether this overestimation was SARS-CoV-2 spike-dependent. HIV-infected participant sera were re-screened with HIV(VSV-G) pseudotypes, replacing the SARS-CoV-2 spike with the VSV-G glycoprotein. Activity against HIV(VSV-G) pseudoviruses was prevalent in HIV-infected participants (75.0% (CI 64.7–85.3%) to 87.9% (CI 80.0-95.8%), per survey) (Fig. 2c), with high median percent neutralisation readings (99.1% (IQR 96.7–99.6%)) (Fig. 2d). Since VSV-G should not be recognised by human sera from Malawi, we hypothesised that the inhibitory activity likely targeted the HIV particles, due to ART drugs affecting luciferase expression from the transfer vector.

**Fig. 3:**
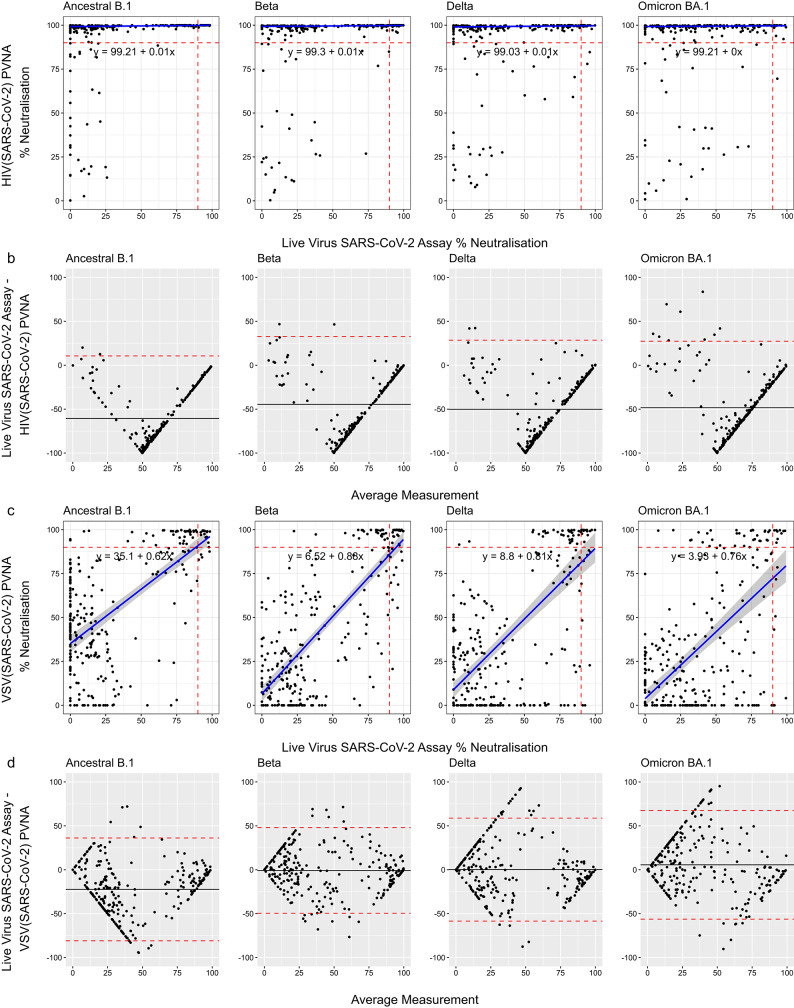
Regression analysis and agreement of the HIV(SARS–CoV–2) PVNA and VSV(SARS–CoV–2) PVNA with a live virus SARS–CoV–2 neutralisation assay in HIV-infected participants. Sera from HIV–infected participants were assessed for neutralising activity against the Ancestral B.1, or variants Beta, Delta, and Omicron BA.1. (**A**) Linear regression analysis between percent neutralisation generated by the HIV(SARS–CoV–2)–based assay and the live virus SARS–CoV–2 assay for each SARS–CoV–2 lineage. Red dotted lines show the 90% cut–off for both assays. Blue line displays the best fit linear regression line with the grey shaded area showing the 95% confidence interval. Text shows the equation of the regression line in the form y = c + mx where m is the gradient. (**B**) Bland–Altman plot showing the agreement between the HIV(SARS–CoV–2)–based assay and the live virus SARS–CoV–2 assay. The black line is the mean difference between the tests for paired samples. The red dotted lines are the 95% limit of agreement. (**C**) Linear regression analysis between percent neutralisation derived using the VSV(SARS–CoV–2)–based assay and the live virus SARS–CoV–2 assays. Red dotted lines show the 90% cut–off for both assays. Blue line displays the best fit correlation with the grey shaded area showing the 95% confidence interval. Blue line displays the best fit linear regression line with the grey shaded area showing the 95% confidence interval. Text shows the equation of the regression line in the form y = c + mx where m is the gradient. (**D**) Bland–Altman plot showing the agreement between the VSV(SARS–CoV–2)–based assay and the live virus SARS–CoV–2 assay. The black line is the mean difference between the tests for paired samples. The red dotted lines are the 95% limit of agreement.

To determine whether the HIV(SARS-CoV-2) or VSV(SARS-CoV-2) assay estimated SARS-CoV-2 neutralisation more accurately in HIV-infected participants, both assays were compared with a SARS-CoV-2 live virus neutralisation assay. For samples from HIV-infected participants, linear regression analysis showed that the HIV(SARS-CoV-2) assay showed no consistency with the live virus assay for all variants, as indicated by the low gradients (in comparison to perfect consistency at y = x, gradient of 1) (Fig. 3). The gradients were: Ancestral B.1–0.01; Beta – 0.01; Delta – 0.01; Omicron BA.1–0.0. Agreement was also poor, with the live virus assay measuring lower values than that for the HIV-based assay (Fig. 3). In contrast, the VSV(SARS-CoV-2) assay demonstrated better consistency with the live virus assay in HIV-infected participants (Ancestral B.1, gradient = 0.62; Beta, 0.88; Delta, 0.81; Omicron BA.1, 0.76) and improved agreement at low and high neutralisation levels (though this was poorer at mid-range values) (Fig. 3). In HIV-uninfected participants, both the HIV and VSV-based assays showed reasonable consistency with the live virus assay (HIV-based and live virus assay: Ancestral B.1, gradient = 0.59; Beta, 0.67; Delta, 0.8; Omicron BA.1, 0.43) (VSV-based and live virus assay: Ancestral B.1, gradient = 0.51; Beta, 0.81; Delta, 0.67; Omicron BA.1, 0.52) (Supplementary Fig. 2). The HIV(SARS-CoV-2) assay showed good agreement with the live virus assay at low and high neutralisation values, but less so at mid-range measurements (Supplementary Fig. 2). The VSV(SARS-CoV-2) assay had poor agreement across the range of measurements (Supplementary Fig. 2).

### Interference with the MLV-based pseudovirus system detected

**Fig. 4:**
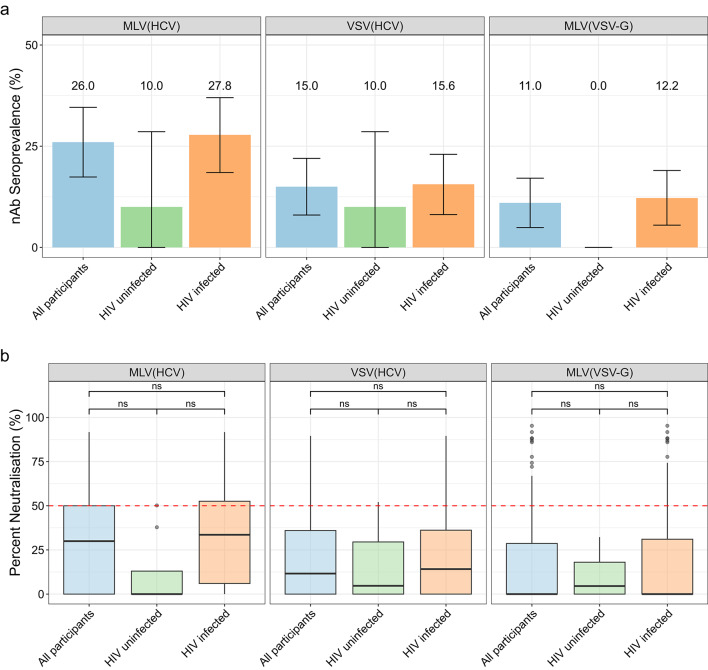
Neutralising activity in sera from the UK cohort, assessed using MLV(HCV), VSV(HCV) and MLV(VSV-G) PVNAs. Samples were grouped into “all participants” (*n* = 100), “HIV uninfected” (*n* = 10), and “HIV infected” (*n* = 90). (**a**) Neutralising antibody (nAb) seroprevalence estimates in all participants, HIV uninfected and HIV infected, measured using MLV(HCV), VSV(HCV) and MLV(VSV-G). Error bars represent 95% confidence intervals, with percentage values shown. (**b**) Percent neutralisation in all participants, HIV uninfected and HIV infected, measured using MLV(HCV), VSV(HCV) and MLV(VSV-G) PVNAs. Box plots display the median and interquartile range (IQR). Red dashed line shows the 50% cut-off. Statistical significance was determined using the Wilcoxon rank-sum test: ns - not significant, *p < 0.05, **p < 0.01, *** *p* < 0.001.

The UK cohort comprised of patients with acute HCV infections (*n* = 100) who were tested for neutralising antibodies against HCV. Within this cohort, estimated HCV neutralising antibody prevalence measured by MLV(HCV) PVNA was 26.0% (CI 17.4–34.6%), with a median percent neutralisation of 30.0% (IQR 0.0-50.1%) for all participants (Fig. 4). When stratifying by HIV status, we found that among HIV-infected participants, estimated HCV neutralising antibody seroprevalence was similar to that observed in all participants at 27.8% (CI 18.5–37.0%) (*p* = 0.91, Chi-sq.), with median percent neutralisation also being similar (33.6% (IQR 6.0-52.6%)) (*p* = 1.0, Wilcoxon) (Fig. 4). HIV-uninfected participants had an estimated HCV neutralising antibody seroprevalence of 10.0% (CI − 8.6–28.6%) (not significantly different from HIV-infected, *p* = 1.0, Chi-sq.) (Fig. 4a). There was also no significant difference in median percent neutralisation when comparing those HIV-uninfected participants (0.0% (IQR 0.0–13.0%)) with those HIV-infected (33.6% (IQR 6.0-52.6%)) (*p* = 0.060, Wilcoxon) (Fig. 4b).

To determine whether the MLV-based system was overestimating HCV neutralisation in the UK cohort, the samples were re-tested using VSV(HCV) pseudotypes. When comparing the results from the VSV(HCV) assay and the MLV(HCV) assay in HIV-infected participants, estimated seroprevalence was 27.8% (CI 18.5–37.0%) with the MLV(HCV) assay, decreasing to 15.6% (CI 8.1–23.0%) when measured with the VSV(HCV) assay (Fig. 4a). This matched the trend in all participants (Fig. 4a). However, in the HIV-uninfected subgroup, seroprevalence was consistent when using both the MLV(HCV) and VSV(HCV) assays at 10% (CI − 8.6–28.6%) (Fig. 4a). Median percent neutralisation measures were also lower using the VSV(HCV) assay compared with the MLV(HCV) assay in all participants (11.6% IQR 0.0–36.0 vs. 30.0% IQR 0.0–50.0, respectively (*p* = 0.014) and those HIV-infected (14.1% IQR 0.0-36.2 vs. 33.5% IQR 6.0-52.6, respectively (*p* = 0.0054) (Fig. 4b). When testing the UK cohort samples using MLV(VSV-G) pseudotypes, activity against the MLV system (independent of surface protein) was detected in 12.2% (CI 5.5–19.0%) of people living with HIV (Fig. 4a). No inhibitory activity was observed in HIV-uninfected participants (prevalence 0.0%), confirming likely interference with the MLV-based pseudotypes when testing HIV-infected participant sera (Fig. 4a).

### Integrase inhibitors interfere with retroviral-based pseudotype systems

We next investigated whether differences in neutralising activity was due to HIV-infected participant sera interfering with HIV particle gene expression post-infection, possibly from residual ART drugs^20,21^. In the Malawi cohort, 79 of the 96 HIV-infected participants (82.3%) reported ART use. Percent neutralisation measured by HIV(SARS-CoV-2) PVNA was similar in those on ART compared with those not on ART, with the only significant difference being observed for the Delta variant - those on ART had higher percent neutralisation than those not on ART (*p* = 0.018, Wilcoxon) (Supplementary Fig. 3). No differences were observed for VSV(SARS-CoV-2) and HIV(VSV-G) pseudotypes stratified by ART use (Supplementary Fig. 3). We restricted analysis to Survey 1 only as self-reported ART receipt was only documented at this survey, however, no differences between those on ART and not on ART were observed for any of the assays (Supplementary Fig. 4).

**Fig. 5:**
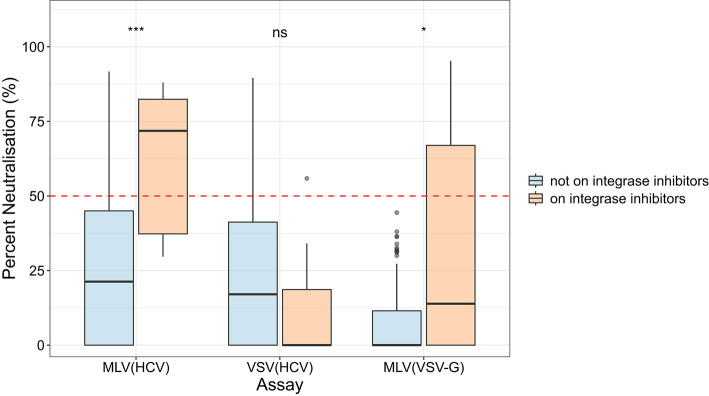
Neutralising activity in sera from the HIV-infected participants of the UK cohort stratified by whether they are receiving integrase inhibitors (*n* = 20) or not (*n* = 62). 8 HIV-infected participants excluded as their ART status/integrase inhibitor use was unknown. Percent neutralisation using the different assay systems - MLV(HCV), MLV(VSV-G) and VSV(HCV), stratified by integrase inhibitor use. Box plots display the median and interquartile range (IQR). Red dashed line shows the 50% cut-off. Statistical test used was Wilcoxon rank sum test: ns - not significant, *p < 0.05, **p < 0.01, *** *p* < 0.001.

Results from the UK cohort (59 of the HIV-infected individuals on ART, 25 not on ART, 6 unknown) showed that those on ART had significantly higher median percent neutralisation compared with those not on ART using the MLV(VSV-G) assay only (0.7% IQR 0.0-31.3% vs. 0.0% IQR 0.0–0.0% respectively) (*p* = 0.033, Wilcoxon) (Supplementary Fig. 5). No significant difference was observed for the MLV(HCV) and VSV(HCV) assays (*p* = 1.0, Wilcoxon). The ART regimen was known for 57 of 59 HIV-infected individuals on ART, of whom 20 (35.1%) were receiving integrase inhibitors. We observed that median percent neutralisation measured using MLV(HCV) pseudotypes was substantially higher in those on integrase inhibitors (71.8%, IQR 37.3–82.4%) compared with those not (21.3%, IQR 0.0–45.0%) (*p* = 5.7 × 10^− 5^, Wilcoxon) (Fig. 5). Moreover, those on integrase inhibitors had significantly lower median percent neutralisation measured using the VSV(HCV) assay (0.0%, IQR 0.0-18.6%) compared with the MLV(HCV) assay (71.8%, IQR 37.3–82.4%) (*p* = 1.9 × 10^− 6^, Wilcoxon), while there was no significant difference for individuals not on integrase inhibitors (*p* = 1.0, Wilcoxon) (Fig. 5). Median percentage neutralisation using the MLV(VSV-G) assay was also higher in those on integrase inhibitors (13.9%, IQR 0.0-66.9%) compared with those not (0.0%, IQR 0.0-11.5%) (*p* = 0.024, Wilcoxon) (Fig. 5). Notably, all of those positive using the MLV(VSV-G) assay (*n* = 11) were receiving integrase inhibitors (all dolutegravir) (Supplementary Table 2). Nine additional participants who were receiving integrase inhibitors did not test positive with the MLV(VSV-G) assay (raltegravir, *n* = 3; dolutegravir, *n* = 6).

**Fig. 6:**
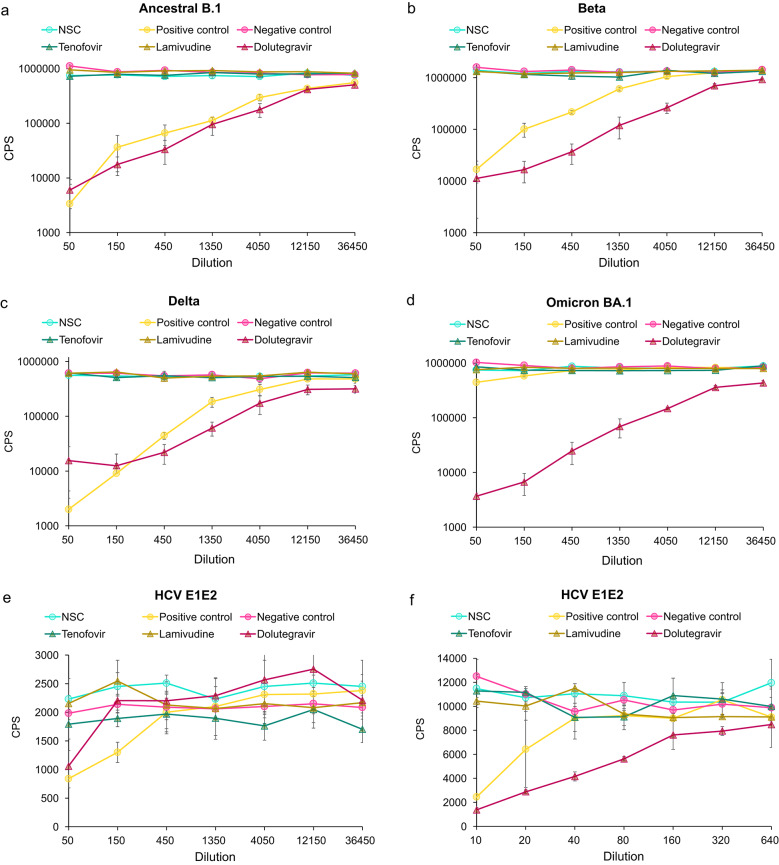
Effect of three antiretroviral drugs (tenofovir, lamivudine and dolutegravir) on the HIV(SARS-CoV-2) and MLV(HCV) PVNAs. (**a**-**d**) Luciferase activity (counts per second (CPS)) for HIV(SARS-CoV-2) PVNA at increasing dilution (1 in 50 to 1 in 36,450) of the no serum control (NSC), positive control serum, negative control serum, tenofovir (326 ng/ml), lamivudine (2,000 ng/ml) and dolutegravir (4,150 ng/ml). HIV(SARS-CoV-2) pseudotypes bearing the (**a**) Ancestral B.1 virus, (**b**) Beta, (**c**) Delta, or (**d**) Omicron BA.1 spike proteins. (**e**) Luciferase activity (CPS) for MLV(HCV) PVNA at increasing dilution (1 in 50 to 1 in 36,450) of the no serum control (NSC), positive control, negative control, tenofovir (326 ng/ml), lamivudine (2,000 ng/ml) and dolutegravir (4,150 ng/ml). (f) Luciferase activity (CPS) for MLV(HCV) PVNA at increasing dilution (1 in 10 to 1 in 640) of the no serum control (NSC), positive control, negative control, tenofovir (326 ng/ml), lamivudine (2,000 ng/ml) and dolutegravir (4,150 ng/ml). We plot the mean measure with error bars displaying the standard deviation of the triplicates.

We then tested three antiretroviral drugs—tenofovir and lamivudine (NRTIs) and dolutegravir (integrase inhibitor)—for interference with the retrovirus-based PVNAs. For the HIV(SARS-CoV-2) pseudotypes, dolutegravir interfered, reducing counts by over 90% for all variants (Fig. 6a to d). The maximum dilution at which counts were reduced by 90% (90% titre) was: Ancestral B.1–1,386, Beta–1,078, Delta–956, and Omicron BA.1–2,316. Note that the SARS-CoV-2 positive control sera did not neutralise Omicron BA.1 virus. This is due to the pooled control samples being from Ancestral B.1 infected individuals (collected March-May 2020) – antibodies generated against Ancestral B.1 very poorly cross neutralise Omicron as it evades immunity^10^. For the MLV(HCV) pseudotypes, dolutegravir interfered, reducing the counts by at least 50% – 50% titre of 52 for the original dilution range (Fig. 6e); 50% titre of 71 when introducing lower dilution points (Fig. 6f). Tenofovir and Lamivudine did not inhibit either pseudotype assays (Fig. 6).

To confirm that the presence of residual drugs in the sera were interfering with the assays, we isolated IgG from Malawi and UK cohort serum samples that were thought to be interfering (discrepancy in retrovirus-based vs. VSV-based pseudovirus assay results). Note that for the Malawi cohort this included individuals that reported being “not on ART”. This IgG isolation removed the drugs from the samples, allowing us to test for neutralising IgG responses to the viruses, without potential drug interference. For the Malawi cohort, in both participants receiving ART and not receiving ART, isolated IgG had lower percent neutralisation of HIV(SARS-CoV-2) than the serum samples against all variant (for those on ART: *p* = 2.48 × 10^− 27^ – all variants; for those not on ART: *p* = 1.30 × 10^− 4^ for Ancestral B.1, Beta, and Delta; *p* = 5.48 × 10^− 4^ for Omicron BA.1). No IgG samples (including those from individuals who reported not receiving ART) were considered positive using the HIV(SARS-CoV-2) assay (i.e. >90% neutralisation) (Supplementary Fig. 6). Notably, there were also differences between the results of VSV(SARS-CoV-2) with serum samples and HIV(SARS-CoV-2) with IgG samples for all variants, likely due to the differing sensitivities of these assays (Supplementary Fig. 6).

For the UK cohort, we observed that isolated IgG samples had far lower percent neutralisation of MLV(HCV) (0.0%, IQR 0.0-9.9%) in comparison with the serum samples (84.3%, IQR 77.1–86.2%) (*p* = 0.0014), showing that isolating IgG removes the interference effect (Supplementary Fig. 7). Furthermore, no significant differences in percent neutralisation were observed between the IgG samples tested with MLV(HCV) and the serum samples tested with VSV(HCV) (*p* = 1.0) (Supplementary Fig. 7).

Based on these analyses, the mechanism of interference is likely integrase inhibitors blocking integration of the luciferase gene and therefore preventing expression of luciferase from the retroviral-based vector when the pseudovirus infects the cell (Supplementary Fig. 8).

## Discussion

To monitor protective immunity to viruses in participant samples, the rapid, sensitive and specific measurement of neutralising antibody activity is required. Retrovirus-based assays are routinely used for SARS-CoV-2^24,25^ and HCV research^26^. In this study, the use of VSV-based control assays demonstrated that the “neutralising activity” using retrovirus-based assays in sera from people living with HIV was not entirely SARS-CoV-2 spike or HCV E1E2 protein specific, but rather also reflected inhibition of luciferase expression from the HIV- or MLV-based vector. The interfering activity was due to residual integrase inhibitors (specifically, dolutegravir) in samples from participants receiving ART.

For SARS-CoV-2, live virus neutralisation assays offered a “gold standard” comparison to the pseudotype assays^25^. In HIV-infected participants (Malawi cohort), the live virus assay showed poor consistency with the HIV(SARS-CoV-2) PVNA, but a strong consistency with the VSV(SARS-CoV-2) PVNA, making VSV-based PVNAs a preferable approach in HIV-prevalent populations. Agreement was weaker at mid-range, where immunoassays typically show greater variation^27,28^, as pipetting/dilution errors disproportionally affected results in this range.

We assessed whether ART components caused the assay interference, as reported previously with HIV(SARS-CoV-2) PVNAs^20,21^. With the Malawi cohort, we observed no significant differences based on ART status (except for Delta variant). ART status was only reported at Survey 1 and therefore may be inaccurate at subsequent study surveys. However, when analysing Survey 1 data separately, we again noted no significant differences based on ART status. For the UK cohort, we observed a significance difference by ART status when using the MLV(VSV-G) assay, but not for the MLV(HCV) or the VSV(HCV) system. This lack of significance with the Malawi cohort could be the result of limited participants who were not receiving antiretrovirals, reducing the power in statistical analysis. However, it should also be noted that ART receipt was self-reported by individuals and may therefore be inaccurate.

Among the UK cohort, we had details on the ART regimen of participants, allowing us to determine that samples from participants receiving integrase inhibitors were those associated with assay interference. However, assay interference was not detected in all participants receiving integrase inhibitors, likely because the drug concentrations in their serum were too low to affect the pseudovirus system. When testing the antiretroviral drugs directly against the pseudotypes, dolutegravir interfered with both retrovirus-based PVNAs, making the presence of integrase inhibitors in sera the likely source of interference. This has not been previously described for MLV-based PVNAs, though it has been documented that anti-HIV integrase inhibitors (specifically, raltegravir) are effective against MLV^29^.

When testing isolated IgG from samples with suspected interference in both cohorts (including Malawian participants who report not receiving ART), percent neutralisation was significantly lower using the IgG compared with the serum samples, with all IgG samples testing negative. This confirms that it is the presence of residual drugs in sera that interferes with the testing. It also provides an additional strategy for testing samples with suspected interference; however, it should be considered than other types of antibodies may have neutralising effects and restricting to only observing the influence of IgG limits the analysis of neutralising immunity. Notably, when testing isolated IgG from participants who were “not on ART” but had discrepancies in their retrovirus-based and VSV-based testing (Malawi cohort), all of these IgG samples were negative (including Survey 1 samples), suggesting that the reporting of ART receipt is inaccurate throughout the study.

The overestimation of neutralising antibodies due to interference was initially observed to be less pronounced in the UK cohort. This was explained by a smaller proportion of the UK cohort being on ART, of whom only 35.1% were receiving integrase inhibitors. Integrase inhibitors are commonly used in HIV and HCV co-infected patients because they do not interact with new directing acting antiviral (DAA) HCV therapies^30^. However, cohort recruitment began in 2005, before the first integrase inhibitors were approved (October 2007)^31^.

Numerous studies have likely inadvertently overestimated neutralising activity in HIV-infected populations. Studies that use retrovirus-based PVNA to characterise neutralising antibody responses in either epidemiological studies or vaccine trials for viruses such as Lassa^32^ and Ebola^33^ (which transmit in HIV prevalent regions) should be cautious in their analyses. Studies that excluded HIV-infected participants because of this interference will not be accurately representing the broader demographic. The exclusion prevents comparison of neutralisation between HIV-infected and HIV-uninfected individuals.

This study was limited in that HIV status and ART use were self-reported and ART regimen data were unavailable for the Malawi cohort. Additionally, we did not test whether protease inhibitors or nucleoside/non-nucleoside reverse transcriptase inhibitors interfere with the retroviral-based assays. Protease inhibitors are less likely to have an effect on retroviral pseudovirus assays as protease is not involved in the gene transfer process in target cells. As described^21^, both nucleoside and non-nucleoside reverse transcriptase inhibitors are unlikely to interfere as they are administered as prodrugs which require phosphorylation to their active state.

In summary, integrase inhibitor presence in sera from people living with HIV interferes with retrovirus-based PVNAs, resulting in inaccurate results in population surveys where the use of integrase inhibitors is prevalent, and HIV-status and ART usage may be unknown. Researchers should instead use alternative systems such as VSV-based PVNA. Caution is advised when interpreting neutralisation data from participants who may be in receipt of integrase inhibitors.

## Methods

### Ethics statement

The Malawi cohort ethical approval was provided by the Malawi College of Medicine Research Ethics Committee (P11/20/3177) and the University of Glasgow College of Medicine, Veterinary and Life Sciences Research and Ethics Committee (200200056). The UK cohort ethical approval was provided by the Riverside Research Ethics Committee, London (5/Q0401/17) and the West of Scotland Research Ethics Committee, Glasgow (12/WS/0002). The study was conducted in accordance with the relevant guidelines and regulations, and adhered to the principles of the Declaration of Helsinki.

### Serum samples and participant metadata

The Malawi cohort included individuals aged *≥* one year from randomly selected households in the Karonga Health and Demographic Surveillance Site and Area 25, Lilongwe (*n* = 2,005)^23^. Written consent was obtained from all participants or their guardians (if < 15 years old or a vulnerable adult). Venous blood was obtained at four three-monthly timepoints (Surveys 1–4), February 2021-April 2022 (Supplementary Fig. 5). This analysis included all adults plus 50% of children (from whom only a small blood volume was collected), provided we received at least one sample, *n* = 1,876 (Survey 1, *n* = 1,515; Survey 2, *n* = 1,322; Survey 3, *n* = 1,215; Survey 4, *n* = 1,123) (Supplementary Table 3). Among these, 5.1% (*n* = 96) were people living with HIV (Survey 1, *n* = 83; Survey 2, *n* = 77; Survey *n* = 3, 68; Survey 4, *n* = 66). Participant characteristics data were collected, including self-reported HIV status and self-reported ART use, at Survey 1 only (Supplementary Table 3).

The UK cohort consisted of 100 individuals with acute HCV infection, of whom 90 were people living with HIV, recruited prospectively from London (St Mary’s Acute HCV cohort)^34^ and Glasgow (Acute HCV UK study), 2005–2019. Informed consent was obtained from all participants. Blood plasma samples and metadata were collected, including HIV status and ART use, as well as ART regimen details (Supplementary Table 3). All serum samples for both the Malawi and UK cohort were heat treated at 56^o^C before being tested.

### Neutralisation assays

#### Malawi cohort

The Malawi cohort samples were tested with four assays, as in the Supplementary Methods. Briefly, SARS-CoV-2 neutralisation was measured using HIV(SARS-CoV-2) pseudoviruses bearing different spike proteins on HIV particles (Supplementary Table 4). For (self-reported) HIV-infected participant samples and a representative, randomly selected (self-reported) HIV-uninfected subset (*n* = 180), VSV(SARS-CoV-2) pseudotypes bearing the SARS-CoV-2 spike proteins on VSV particles determined the neutralisation specificity^35^. HIV(VSV-G) pseudoviruses bearing the VSV-G protein on HIV particles determined if neutralising activity in HIV-infected participants was outer protein (SARS-CoV-2 spike)-dependent. VSV is largely absent in Africa and Europe, making VSV-G a useful serological control^36^. Live virus neutralisation assays were used as a “gold standard” comparison. For the Malawi cohort testing, samples with percent neutralisation > 90% were classed as positive.

#### UK cohort

The UK cohort samples were tested with three assays, as described in the Supplementary Methods. Briefly, HCV neutralisation was measured using MLV(HCV) pseudotypes bearing the E1E2 protein of HCV genotype 1a on MLV particles. VSV(HCV) pseudotypes bearing the E1E2 protein on VSV particles determined the neutralising activity specificity. MLV(VSV-G) pseudotypes bearing the VSV-G protein on MLV particles determined if the neutralisation was outer protein (E1E2)-dependent. For the UK cohort testing, samples with percent neutralisation > 50% were classed as positive.

### ART testing

To determine if non-specific inhibitory activity in the sera was due to the presence of residual antiretroviral drugs, we tested antiretroviral drugs for interference with the HIV(SARS-CoV-2) and MLV(HCV)-based assays. The drugs tested were those commonly used as first-line ART regimen in Malawi and the UK during the study period: tenofovir and lamivudine (nucleoside reverse transcriptase inhibitors, NRTIs), and dolutegravir (integrase inhibitor)^37^. The drugs were diluted to their Cmax (maximum blood serum concentration) in distilled water: tenofovir-326 ng/ml (physiological range: 64–326 ng/ml)^38^; lamivudine-2,000 ng/ml (range: 40 − 2,000 ng/ml)^39^; and dolutegravir-4,150 ng/ml (range: 2,120-4,150 ng/ml)^40^. The drugs and controls (Supplementary Methods) were serially diluted in complete DMEM from 1 in 50 to 1 in 36,450 (3-fold steps) before incubation with the virus and cells (as per assay requirements, Supplementary Methods). For MLV(HCV) interference testing, we performed an additional assay where we reduced the drug and control dilutions, using a dilution range of 1 in 10 to 1 in 640 (2-fold steps).

### IgG purification

IgG was purified from serum samples with suspected assay interference, i.e. those with a discrepancy in retrovirus-based vs. VSV-based assay results. For the UK cohort, this included samples from participants who tested neutralisation positive with the MLV(HCV) assay, but negative with the VSV(HCV) assay. For the Malawi cohort, this included samples from participants who tested neutralisation positive with the HIV(SARS-CoV-2) assay, but negative with the VSV(SARS-CoV-2) assay. IgG was isolated using the Cytiva protein G high performance SpinTrap™ columns, as per manufacturer guidelines.

### Data analyses

Microsoft Excel and R version 4.0.5^41^ (ggplot2, ggsignif and ggpubr, packages) were used for data analyses and visualisations. For seroprevalence percentages, 95% binomial confidence intervals (CI) were reported to estimate the uncertainty around the proportion of seropositive individuals. A two-proportion chi-squared test (two-tailed) was used to compare the proportion of seropositive individuals between groups, as this test is appropriate for categorical data and evaluates whether observed differences in proportions are statistically significant. For percent neutralisation measures, the median and interquartile range (IQR) were used as the data were not normally distributed. A two-tailed Wilcoxon rank-sum test was chosen because it is a non-parametric test that compares distributions without assuming normality, making it more appropriate for skewed data. When making multiple individual statistical comparisons, a Bonferroni correction was applied to control for type 1 errors and reduce the likelihood of false positives.

For comparisons to the live virus assay (SARS-CoV-2 analysis), robust linear regression analysis (robustbase package) was performed. Traditional correlation analysis was inappropriate as the data were not normally distributed and contained outliers that could skew results when using the non-parametric correlation methods. Robust linear regression, a method that reduces the influence of outliers and does not assume normality, was selected to provide a more reliable analysis. The equation of the regression line was presented, and the gradient was compared to that of the line y = x (which represents perfect agreement, where the gradient equals 1). Extensive deviations from a gradient of 1 indicate systematic differences between the two assays. Additionally, Bland-Altman plots were used to evaluate the agreement between assays by analysing the differences between paired measurements across the range of values. An alpha level of 0.05 was used for statistical comparisons, meaning that p-values < 0.05 were considered statistically significant.

## Electronic supplementary material

Below is the link to the electronic supplementary material.

Supplementary Material 1

## Data Availability

The data that support the findings of this study are available from the Malawi Epidemiology and Intervention Research Unit (MEIRU) but restrictions apply to the availability of these data, which were used under license for the current study, and so are not publicly available. Data are however available from the authors upon reasonable request and with permission of MEIRU. Data requests should be sent to Mhairi J. McCormack – m.mccormack.1@research.gla.ac.uk.

## References

[1] Khoury, D. S. et al. Neutralizing antibody levels are highly predictive of immune protection from symptomatic SARS-CoV-2 infection. *Nat. Med.* **27**, 1205–1211 (2021). 34002089 10.1038/s41591-021-01377-8

[2] Cromer, D. et al. Neutralising antibody titres as predictors of protection against SARS-CoV-2 variants and the impact of boosting: a meta-analysis. *Lancet Microbe*. **3**, e52–e61 (2022). 34806056 10.1016/S2666-5247(21)00267-6 PMC8592563

[3] Feng, S. et al. Correlates of protection against symptomatic and asymptomatic SARS-CoV-2 infection. *Nat. Med.* **27**, 2032–2040 (2021). 34588689 10.1038/s41591-021-01540-1 PMC8604724

[4] Gilbert, P. B. et al. Immune correlates analysis of the mRNA-1273 COVID-19 vaccine efficacy clinical trial. *Scienc* **375**, 43–50 (2022). 10.1126/science.abm3425 PMC9017870 34812653

[5] Fong, Y. et al. Immune correlates analysis of the ENSEMBLE single Ad26.COV2.S dose vaccine efficacy clinical trial. *Nat. Microbiol.* **7**, 1996–2010 (2022). 36357712 10.1038/s41564-022-01262-1 PMC10166187

[6] Fong, Y. et al. Immune correlates analysis of the PREVENT-19 COVID-19 vaccine efficacy clinical trial. *Nat. Commun.* **14**, 331 (2023). 36658109 10.1038/s41467-022-35768-3 PMC9851580

[7] Wall, E. C. et al. Neutralising antibody activity against SARS-CoV-2 VOCs B.1.617.2 and B.1.351 by BNT162b2 vaccination. *Lancet* **397**, 2331–2333 (2021). 34090624 10.1016/S0140-6736(21)01290-3 PMC8175044

[8] D’Apice, L. et al. Comparative analysis of the neutralizing activity against SARS-CoV-2 Wuhan-Hu-1 strain and variants of concern: performance evaluation of a pseudovirus-based neutralization assay. *Front. Immunol.* **13**, 981693 (2022). 36225911 10.3389/fimmu.2022.981693 PMC9549111

[9] Davis, C. et al. Reduced neutralisation of the Delta (B.1.617.2) SARS-CoV-2 variant of concern following vaccination. *PLoS Pathog*. **17**, e1010022 (2021). 34855916 10.1371/journal.ppat.1010022 PMC8639073

[10] Willett, B. J. et al. SARS-CoV-2 Omicron is an immune escape variant with an altered cell entry pathway. *Nat. Microbiol.* **7**, 1161–1179 (2022). 35798890 10.1038/s41564-022-01143-7 PMC9352574

[11] Hu, S. et al. Correlating IgG levels with neutralising antibody levels to indicate clinical protection in healthcare workers at risk during a measles outbreak. *Viruses* **14**, 8 (2022). 10.3390/v14081716 PMC9415042 36016338

[12] Ferrara, F. & Temperton, N. Pseudotype neutralization assays: from laboratory bench to data analysis. *Methods Protoc.* **1**, 1 (2018). 10.3390/mps1010008 PMC6526431 31164554

[13] Wang, S. et al. Establishment of a pseudovirus neutralization assay based on SARS-CoV-2 S protein incorporated into lentiviral particles. *Biosaf. Health*. **4**, 38–44 (2022). 35005601 10.1016/j.bsheal.2021.12.006 PMC8721934

[14] Nie, J. et al. Quantification of SARS-CoV-2 neutralizing antibody by a pseudotyped virus-based assay. *Nat. Protoc.* **15**, 3699–3715 (2020). 32978602 10.1038/s41596-020-0394-5

[15] Tsai, W. Y. et al. A real-time and high-throughput neutralization test based on SARS-CoV-2 pseudovirus containing monomeric infrared fluorescent protein as reporter. *Emerg. Microbes Infect.* **10**, 894–904 (2021). 33929934 10.1080/22221751.2021.1925163 PMC8143625

[16] Li, Q. et al. Current status on the development of pseudoviruses for enveloped viruses. *Rev. Med. Virol.* **28**, 1 (2018). 10.1002/rmv.1963 PMC7169153 29218769

[17] Wibmer, C. K. et al. SARS-CoV-2 501Y.V2 escapes neutralization by South African COVID-19 donor plasma. *Nat. Med.* **27**, 622–625 (2021). 33654292 10.1038/s41591-021-01285-x

[18] Madhi, S. A. et al. Durability of ChAdOx1 nCoV-19 (AZD1222) vaccine and hybrid humoral immunity against variants including Omicron BA.1 and BA.4 6 months after vaccination (COV005): A post-hoc analysis of a randomised, phase 1b-2a trial. *Lancet Infect. Dis.* **23**, 295–306 (2023). 36273491 10.1016/S1473-3099(22)00596-5 PMC9584570

[19] Madhi, S. A. et al. Efficacy of the ChAdOx1 nCoV-19 Covid-19 baccine against the B.1.351 bariant. *N Engl. J. Med.* **384**, 1885–1898 (2021). 33725432 10.1056/NEJMoa2102214 PMC7993410

[20] Neerukonda, S. N. et al. Establishment of a well-characterized SARS-CoV-2 lentiviral pseudovirus neutralization assay using 293T cells with stable expression of ACE2 and TMPRSS2. *PLoS One*. **16**, e0248348 (2021). 33690649 10.1371/journal.pone.0248348 PMC7946320

[21] De La Torre-Tarazona, E. et al. Treatment with integrase inhibitors alters SARS-CoV-2 neutralization levels measured with HIV-based pseudotypes in people living with HIV. *J. Med. Virol.* **95**, e28543 (2023). 36727646 10.1002/jmv.28543

[22] Mishra, N. et al. Cross-neutralization of SARS-CoV-2 by HIV-1 specific broadly neutralizing antibodies and polyclonal plasma. *PLOS Pathog*. **17**, e1009958 (2021). 34559854 10.1371/journal.ppat.1009958 PMC8494312

[23] Banda, L. et al. Characterizing the evolving SARS-CoV-2 Seroprevalence in urban and rural Malawi between February 2021 and April 2022: A population-based cohort study. *Int. J. Infect. Dis.* **137**, 118–125 (2023). 38465577 10.1016/j.ijid.2023.10.020 PMC10695832

[24] Manali, M. et al. SARS-CoV-2 evolution and patient immunological history shape the breadth and potency of antibody-mediated immunity. *J. Infect. Dis.* **227**, 40–49 (2022). 35920058 10.1093/infdis/jiac332 PMC9384671

[25] Bewley, K. R. et al. Quantification of SARS-CoV-2 neutralizing antibody by wild-type plaque reduction neutralization, microneutralization and pseudotyped virus neutralization assays. *Nat. Protoc.* **16**, 3114–3140 (2021). 33893470 10.1038/s41596-021-00536-y

[26] Bartosch, B. et al. Infectious hepatitis C virus pseudo-particles containing functional E1-E2 envelope protein complexes. *J. Exp. Med.* **197**, 633–642 (2003). 12615904 10.1084/jem.20021756 PMC2193821

[27] Biosensis Data analysis for ELISA assays. (2016). https://www.biosensis.com/documents/enhancedinfo/Technical-Note-2-ELISA-Data-Analysis.pdf

[28] Nguyen, T. T. et al. Robust dose-response curve Estimation applied to high content screening data analysis. *Source Code Biol. Med.* **9**, 27 (2014). 25614758 10.1186/s13029-014-0027-x PMC4279979

[29] Beck-Engeser, G. B. et al. Early onset of autoimmune disease by the retroviral integrase inhibitor raltegravir. *Proc. Natl. Acad. Sci. USA.* 106, 20865–20870 (2009). 10.1073/pnas.0908074106 PMC2791572 19923437

[30] Feng, H. P. et al. Assessment of drug interaction potential between the HCV direct-acting antiviral agents elbasvir/grazoprevir and the HIV integrase inhibitors raltegravir and dolutegravir. *J. Antimicrob. Chemother.* **74**, 710–717 (2018). 10.1093/jac/dky465 30541077

[31] Merck Merck receives FDA approval for ISENTRESS^®^ (raltegravir), in combination with other antiretroviral agents, for the treatment of HIV-1 infection in newborns weighing at least 2 kg. (2017). https://www.merck.com/news/merck-receives-fda-approval-for-isentress-raltegravir-in-combination-with-other-antiretroviral-agents-for-the-treatment-of-hiv-1-infection-in-newborns-weighing-at-least-2-kg/

[32] Cosset, F. L. et al. Characterization of Lassa virus cell entry and neutralization with Lassa virus pseudotypes. *J. Virol.* **83**, 3228–3237 (2009). 19153226 10.1128/JVI.01711-08 PMC2655582

[33] Luczkowiak, J. et al. Specific neutralizing response in plasma from convalescent patients of Ebola virus disease against the West Africa Makona variant of Ebola virus. *Virus Res.* **213**, 224–229 (2016). 26739425 10.1016/j.virusres.2015.12.019

[34] Thomson, E. C. et al. Predicting spontaneous clearance of acute hepatitis C virus in a large cohort of HIV-1 infected men. *Gut* **60**, 837–845 (2011). 21139063 10.1136/gut.2010.217166 PMC3095479

[35] Logan, N. et al. Efficient generation of vesicular stomatitis virus (VSV)-pseudotypes bearing morbilliviral glycoproteins and their use in quantifying virus neutralising antibodies. *Vaccine* **34**, 814–822 (2016). 26706278 10.1016/j.vaccine.2015.12.006 PMC4742518

[36] Rozo-Lopez, P. et al. Comparison of endemic and epidemic vesicular stomatitis virus lineages in Culicoides sonorensis midges. *Viruses* **14**, 1221 (2022). 35746691 10.3390/v14061221 PMC9230599

[37] Ministry of Health and Population Malawi. Malawi integrated clinical HIV guidelines 5th Edition. (2022). https://dms.hiv.health.gov.mw/dataset/malawi-intergrated-clinical-hiv-guidelines-1st-edition-2022

[38] Electronic Medicines Compendium (EMC). Viread 245 mg film-coated tablets – Summary of Product Characteristics (SmPC). (2024). https://www.medicines.org.uk/emc/product/1615/smpc

[39] Electronic Medicines Compendium (EMC). Epivir 150 mg film-coated tablets – Summary of Product Characteristics (SmPC). (2025). https://www.medicines.org.uk/emc/product/8048/smpc

[40] U.S. Food and Drug Administration (FDA). Tivicay (dolutegravir) Prescribing Information. (2024). https://www.accessdata.fda.gov/drugsatfda_docs/pepfar/210237PI.pdf

[41] RStudio Team R. RStudio: integrated development environment for R. 2023.09.1 + 494. RStudio, PBC, (2023).

