# Supplementary material for: Retrovirus-based pseudotyped virus neutralisation assays overestimate neutralising activity in sera from participants receiving integrase inhibitors
Source: Sci Rep. 2025 Aug 5;15:28580. doi: 10.1038/s41598-025-11362-7 (PMC12325791; doi:10.1038/s41598-025-11362-7)
Supplement: Supplementary file 1 — Supplementary Material 1 [file 41598_2025_11362_MOESM1_ESM.pdf]

Supplementary Materials

Retrovirus-based pseudotyped virus neutralisation assays overestimate neutralising activity in sera from participants receiving integrase inhibitors

Authors:

*Mhairi J. McCormack BSc (Hons)[1], m.mccormack.1@research.gla.ac.uk
Patawee Asamaphan PhD[1], patawee.asamaphan@glasgow.ac.uk
Ellen C. Hughes PhD[1,2], ellen.hughes@liverpool.ac.uk
Louis Ba MSc[3], louis.banda@meiru.mw
Stephen Kasenda MBBS[3], stephen.kasenda@meiru.mw
Chris Davis PhD[1], chris.davis@glasgow.ac.uk
Agnieszka M. Szemiel PhD[1], agnieszka.szemiel@glasgow.ac.uk
Amelia Crampin MSc[3,4], mia.crampin@lshtm.ac.uk
Abena S. Amoah PhD[3,4,6†], a.s.amoah@lumc.nl
Emma C. Thomson PhD[1,4†], emma.thomson@glasgow.ac.uk
Antonia Ho PhD[1†], antonia.ho@glasgow.ac.uk
Brian J. Willett PhD[1†], brian.willett@glasgow.ac.uk

[†]These authors contributed equally to this work

Affiliations:

1. Medical Research Council-University of Glasgow Centre for Virus Research, Glasgow, UK
2. Department of Livestock and One Health, Institute of infection, veterinary and ecological sciences, University of Liverpool, Liverpool, UK
3. Malawi Epidemiology and Intervention Research Unit (MEIRU), Malawi
4. London School of Hygiene and Tropical Medicine, London, UK
5. School of Health and Wellbeing, University of Glasgow, Glasgow, UK
6. Leiden University Medical Center, Leiden, Netherlands

Table of Contents

Supplementary Methods 3

Cells 3

Production of pseudotypes 3

Pseudotypes virus neutralisation assay (PVNA) methods 4

SARS–CoV–2 Live Virus Neutralisation Assay 5

ART testing controls 5

Supplementary Tables........ 6

Supplementary Table 1. Comparisons of SARS-CoV-2 percent neutralisation between all participants and those HIV-infected. 6

Supplementary Table 2. Cross tabulation of integrase inhibitor use and MLV(VSV–G) assay positivity. . 6

Supplementary Table 3. Participant characteristics at baseline for the Malawi and UK cohort. 7

Supplementary Table 4. Spike gene construct mutations for the SARS–CoV–2 variants, relative to the Wuhan–Hu–1 sequence (GenBank: MN908947). 7

Supplementary Figures 8

Supplementary Figure 1. Timing of the four survey periods in comparison with the national daily new cases of laboratory–confirmed COVID–19 in Malawi – data from the Public Health Institute of Malawi. 8

Supplementary Figure 2. Regression analysis and agreement of the HIV(SARS–CoV–2) PVNA and VSV(SARS–CoV–2) PVNA with a live virus SARS–CoV–2 neutralisation assay in HIV-uninfected participants. 9

Supplementary Figure 3. Neutralising activity in sera from HIV–infected participants across all four study surveys (Malawi cohort, n=95) using HIV(SARS–CoV–2), VSV(SARS–CoV–2) and HIV(VSV–G) PVNAs, stratified by whether they report receiving antiretroviral therapy (ART) (not on ART – n=17, on ART – n=78)........ 11

Supplementary Figure 4. Neutralising activity in sera from HIV–infected participants for survey 1 only (Malawi cohort, n=83) using HIV(SARS–CoV–2), VSV(SARS–CoV–2) and HIV(VSV–G) PVNAs, stratified by whether they report receiving antiretroviral therapy (ART) (not on ART – n=13, on ART – n=70) 13

Supplementary Figure 5. Neutralising activity in sera from HIV–infected participants who reported their ART use (UK cohort, n=84) using MLV(HCV), MLV(VSVG) and VSV(HCV) PVNAs, stratified by whether they report receiving antiretroviral therapy (ART) (not on ART – n=25, on ART – n=59). 14

Supplementary Figure 6. Neutralisation of HIV(SARS-CoV-2) and VSV(SARS-CoV-2) by serum and neutralisation of HIV(SARS-CoV-2) by isolated IgG in participants with discrepant neutralisation profiles (Malawi cohort, n=96). 15

Supplementary Figure 7. Neutralisation of MLV(HCV) and VSV(HCV) by serum and neutralisation of MLV(HCV) by isolated IgG in participants with discrepant neutralisation profiles (UK cohort, n=12). .. 16

Supplementary Figure 8. Proposed mechanism of interference with the retroviral–based pseudotypes by integrase inhibitors. 17

References for Supplementary Materials 17

Supplementary Methods

Cells

All cell lines were maintained at 37 °C, 5% CO_2 . Human embryonic kidney 293T cells (HEK293T, produced by stable transduction of HEK293 cells with a plasmid encoding the SV40 large T antigen) were maintained in Dulbecco's modified Eagle's medium (DMEM) supplemented with 10% foetal bovine serum (FBS), 2 mM L–glutamine, 100 µg/ml streptomycin and 100 IU/ml penicillin (complete DMEM) with 400 µg/ml G418. HEK293–ACE2 cells, produced by stable transduction of HEK293 cells with pSCRPSY–hACE2 (a vector carrying the human Angiotensin-Converting Enzyme 2, ACE-2, gene), were maintained in complete DMEM supplemented with 2 µg/ml puromycin. Huh–7 cells (human hepatocellular carcinoma derived cells) and AAT cells (human alveolar adenocarcinoma cells, A549, engineered to overexpress ACE-2 and Transmembrane Protease Serine 2, TMPRSS2) [1] were maintained in complete DMEM.

Production of pseudotypes

HIV(SARS–CoV–2):
HEK293T cells were transfected with 0.15 µg/ml of the appropriate SARS–CoV–2 spike gene expression vector (Ancestral B.1, Beta, Delta, or Omicron BA.1) (codon optimised and synthesised by GenScript) together with 0.1 µg/ml of p8.91 (HIV gag-pol plasmid from Addgene) [2] and 0.1µg/ml of pCSFLW (firefly luciferase plasmid) [3] using 1µl/ml of polyethylenimine transfection agent (PEI, Polysciences, Warrington, USA). The plates were incubated at 37 °C for 48 hours, then the supernatants containing the pseudoviruses were harvested, filtered through a 0·45 µm filter, and stored at −80 °C.

VSV(SARS–CoV–2):
To generate the VSV(SARS–CoV–2) pseudoviruses, 0.5 µg/ml of the appropriate SARS–CoV–2 spike gene expression vectors (Ancestral B.1, Beta, Delta, or Omicron BA.1) (codon optimised and synthesised by GenScript) were combined with 6 µl/ml of polyethylenimine transfection agent (PEI, Polysciences, Warrington, USA), added to HEK293T cells and incubated for four hours at 37 °C. VSV–ΔG luc (encoding the essential VSV genes - apart from the G protein - and the firefly luciferase reporter gene; gifted from Michael Whitt, Memphis, Tennessee, USA) was added to the cells at a multiplicity of infection (MOI) of 0·02 and incubated for one hour at 37 °C. The medium was removed, and the cells were washed three times with phosphate buffered saline (PBS). Complete DMEM was added, and the cells were incubated for 48 hours at 37 °C. Supernatants containing the pseudoviruses were harvested, filtered through a 0·45 µm filter and stored at –80 °C.

HIV(VSV–G):
HEK293T cells were transfected with 0.15 µg/ml of the VSV–G gene expression vector (pMD.G, from Addgene) [4] together with 0.1 µg/ml of p8.91 (HIV gag-pol plasmid from Addgene) [2,3] and 0.1µg/ml of pCSFLW (firefly luciferase plasmid) [2,3] using 1µl/ml of polyethylenimine transfection agent (PEI, Polysciences, Warrington, USA). The plates were incubated at 37 °C for 48 hours, then the supernatants containing the pseudoviruses were harvested, filtered through a 0·45 µm filter, and stored at −80 °C.

MLV(HCV):
HEK293T cells were transfected with 0.3 µg/ml of the E1/E2 plasmid of HCV genotype 1a [5,6], 0.8 µg/ml of the MLV gag/pol plasmid [5,6] and 0.8 µg/ml of the luciferase protein plasmid [5,6], using the Fugene 6 transfection reagent (Promega). The cells were seeded and incubated for 24 hours at

37 °C before transfection. The transfection was then incubated for a further 24 hours before the media was refreshed. After a final 24 hour incubation, supernatants containing the pseudoviruses were harvested, filtered through a 0·45 µm filter and stored at –80 °C.

MLV(VSV–G):
HEK293T cells were transfected with 0.3 µg/ml of the VSV–G gene expression vector (pMD.G plasmid, from Addgene), 0.8 µg/ml of the MLV gag/pol plasmid [5,6] and 0.8 µg/ml of the luciferase protein plasmid [5,6], using the Fugene 6 transfection reagent (Promega). The cells were seeded and incubated for 24 hours at 37 °C before transfection. The transfection was then incubated for a further 24 hours before the media was refreshed. After a final 24 hour incubation, supernatants containing the pseudoviruses were harvested, filtered through a 0·45 µm filter and stored at –80 °C.

VSV(HCV):
To generate the VSV(HCV) pseudoviruses, 0.7 µg/ml of the E1/E2 HCV 1a plasmid was combined with 8.4 µl/ml of polyethylenimine transfection agent (PEI, Polysciences, Warrington, USA), added to HEK293T cells and incubated for four hours at 32 °C. VSV–ΔG luc (encoding the essential VSV genes - apart from the G protein - and the firefly luciferase reporter gene; gifted from Michael Whitt, Memphis, Tennessee, USA) was added to the cells at a multiplicity of infection (MOI) of 0·02 and incubated for one hour at 32 °C. The medium was removed, and the cells were washed three times with phosphate buffered saline (PBS). Complete DMEM was added, and the cells were incubated for 48 hours at 32 °C. Supernatants containing the pseudoviruses were harvested, filtered through a 0·45 µm filter and stored at –80 °C.

Pseudotypes virus neutralisation assay (PVNA) methods

For HIV(SARS–CoV–2), VSV(SARS–CoV–2) and HIV(VSV–G):
Samples were tested in a single dilution screen. Sera were diluted in complete DMEM (1 in 25) and added in duplicate to white 96-well plates. 25µl per well of the specified pseudovirus was added (final serum dilution 1 in 50). HEK293–ACE2 target cells were added and after 48–72 hour incubation at 37 °C, luciferase activity was measured by adding Steadylite Plus chemiluminescence substrate and analysed with a Perkin Elmer EnSight multimode plate reader (Perkin Elmer, Beaconsfield, UK). Samples with percent neutralisation >90% were classed as posit ive.

For MLV(HCV) and MLV(VSV–G):
Samples were tested in a single dilution screen. Samples were diluted in PBS and incubated for one hour with the pseudovirus (final serum dilution 1 in 50). This mixture was added to Huh–7 cells seeded the previous day and incubated at 37 °C for 3 hours. The media was removed and replaced with fresh media before being incubated at 37 °C for 3 days. Cells were washed with PBS, lysed with Go lysis buffer (Promega) and BrightGlo reagent was added at a 1:1 ratio. Luciferase activity was measured using Hidex microplate. For the ART testing against MLV(HCV), luciferase activity was instead measured by adding Steadylite Plus chemiluminescence substrate and analysed with a Perkin Elmer EnSight multimode plate reader (Perkin Elmer, Beaconsfield, UK). Samples with percent neutralisation >50% were classed as positive.

For VSV(HCV):
Samples were tested in a single dilution screen. Sera were diluted in complete DMEM (1 in 25) and added in duplicate to white 96-well plates. Prior to adding the virus, it was incubated with anti–VSV monoclonal antibody (obtained from American Type Culture Collection, ATCC) [7] for one hour at 32 °C to reduce the VSV background. 25µl per well of the specified pseudovirus was added (final serum dilution 1 in 50). Huh–7 target cells were added and after 48 hour incubation at 32 °C, luciferase activity was measured by adding Steadylite Plus chemiluminescence substrate and analysed with a

Perkin Elmer EnSight multimode plate reader (Perkin Elmer, Beaconsfield, UK). Samples with percent neutralisation >50% were classed as positive.

SARS–CoV–2 Live Virus Neutralisation Assay

SARS–CoV–2 England–02 (hCoV–19/England/02/2020, GISAID accession EPI_ISL_407073) was obtained from Public Health England. The Beta (B.1.351) and Delta (B.1.617.2), variant isolates were kindly provided by Wendy Barclay, (GISAID accession numbers: EPI_ISL_770441 and EPI_ISL_1731019, respectively). The Omicron B.1.1.529.1 (BA.1) variant was previously described [8] (GISAID accession number EPI_ISL_10666879). For the Malawi cohort HIV–infected samples and HIV–uninfected participant subset (n=180), the following method was used. AAT cells were seeded at a density of $2x10^4$ cells/well. The serum samples were diluted 1in25 and incubated for 60 to 90 minutes at 37 °C with of SARS–CoV–2 B.1 (D614G), Beta (B.1.351), Delta (B.1.617.2) or Omicron BA.1 (B.1.1.529) variants at an MOI 0·5 or 0·01. The virus and antibody mixtures were applied to cells and incubated for 40–44 hours (B.1, Beta and Delta) or 44–60 hours (Omicron BA.1) before fixing in 8% formaldehyde. The cells were stained with primary sheep anti–N polyclonal antibody (DA103 or DA114, Dundee University) [1] and secondary donkey anti–sheep AlexaFluor 555 (Invitrogen, Cat No. A–21436) or donkey anti–sheep AlexaFluor 488 (Invitrogen, Cat No. A11015) secondary antibodies. A Celigo (Nexcelom Bioscience) imaging cytometer measured fluorescence.

ART testing controls

For the HIV(SARS-CoV-2) testing, the following controls were included: no–serum control (NSC) - complete DMEM; negative control – pre–COVID–19 pandemic pooled serum; positive control – pooled SARS-CoV-2 positive sera. The SARS-CoV-2 positive control sera were residual serum samples from primary and secondary healthcare settings. The samples were collected between March to May 2020 from infected individuals with a confirmed positive PCR test for SARS-CoV-2. Ethical approval was provided by NHS Greater Glasgow and Clyde (GGC) Biorepository (application 550). The SARS-CoV-2 negative control sera was a single individual, pre-pandemic donation from the Scottish National Blood Transfusion Service (SNBTS). Ethical approval for this was covered by SNBTS (NATF 765 10).

For the MLV(HCV) testing, the following controls were included: no-serum control (NSC) – complete DMEM; negative control – HC12 (purified IgG from a healthy control, recruited under the UK cohort ethics - Riverside Research Ethics Committee, London (5/Q0401/17) and the West of Scotland Research Ethics Committee, Glasgow (12/WS/0002)); positive control - AP33 monoclonal antibody gifted from Arvind Patel [9].

Supplementary Tables

Supplementary Table 1. Comparisons of SARS-CoV-2 percent neutralisation between all participants and those HIV-infected. The median (IQR) is provided along with the effect size, and median difference (95% CI).

	Participants	Median (IQR)	Effect size	Median difference (95% CI)
**Ancestral B.1**	All participants	35.2 (10.4-90.2)	··	··
	HIV-uninfected	32.1 (9.2-80.8)	0.035	3.1 (0.7-5.5)
**Beta**	All participants	30.1 (8.0-84.0)	··	··
	HIV-uninfected	28.1 (6.5-69.3)	0.036	2.0 (0.4-3.8)
**Delta**	All participants	26.6 (7.0-80.2)	··	··
	HIV-uninfected	24.3 (5.8-65.4)	0.036	2.3 (0.6-3.8)
**Omicron BA.1**	All participants	23.5 (1.0-56.6)	··	··
	HIV-uninfected	21.7 (0.0-48.9)	0.039	1.8 (0.4-3.5)

IQR – interquartile range; CI – confidence interval.

Supplementary Table 2. Cross tabulation of integrase inhibitor use and MLV(VSV–G) assay positivity. For the 57 participants of the UK cohort that are HIV–infected and receiving ART therapy that we know the regimen details for.

	MLV(VSV–G) assay negative	MLV(VSV–G) assay positive
**Not receiving integrase inhibitors**	37 (64·9%)	0 (0·0%)
**Receiving integrase inhibitors**	9 (15·8%)	11 (19·3%)

MLV – murine leukaemia virus; VSV–G = vesicular stomatitis virus–glycoprotein.

Supplementary Table 3. Participant characteristics at baseline for the Malawi and UK cohort.

	Malawi cohort (n=1,876)	UK cohort (n=100)
**Sampling period**	February 2021–April 2022	January 2005–October 2019
**Median age, years (IQR)**	24·0 (12·8–40·5)	39·0 (32.–45·3)
**Age category (n, %)**	··	··
<15	557 (29·7)	0 (0·0)
15–39	834 (44·5)	51 (51·0)
40–59	351 (18·7)	46 (46·0)
>=60	134 (7·1)	3 (3·0)
**Sex (n, %)**	··	··
Female	1,046 (55·8)	5 (5·0)
Male	830 (44·2)	95 (95·0)
**Comorbidities (n, %)**	··	··
None	1606 (85·6)	22 (22·0)
1	243 (13·0)	25 (25·0)
≥2	27 (1·4)	53 (53·0)
**HIV–infected (n, %)**	96 (5·1)	90 (90·0)
**ART use (among HIV–infected) (n, %)**	··	··
On ART	79 (82·3)	59 (59·0)
Not on ART	17 (17·7)	25 (25·0)
Unknown	0 (0·0)	6 (6·0)
**Receiving integrase inhibitors (among those on ART) (n, %)**	··	··
Yes	NA	20 (33·9)
No	NA	37 (62·7)
Unknown	79 (100·0)	2 (3·4)

IQR – interquartile range; HIV – human immunodeficiency virus; ART – antiretroviral therapy; NA – not applicable.

Supplementary Table 4. Spike gene construct mutations for the SARS–CoV–2 variants, relative to the Wuhan–Hu–1 sequence (GenBank: MN908947).

	Mutations relative to Wuhan–Hu–1 sequence
Ancestral (B.1)	D614G
Beta (B.1.351)	D80A, D215G, L241del, L242del, A243del, K417N, E484K, N501Y, D614G, A701V
Delta (B.1.617.2)	T19R, G142D, Δ156–157, R158G, L452R, T478K, D614G, P681R, D950N
Omicron BA.1 (B.1.1.529)	A67V, Δ69–70, T95I, G142D/Δ143–145, Δ211/L212I, ins214EPE, G339D, S371L, S373P, S375F, K417N, N440K, G446S, S477N, T478K, E484A, Q493R, G496S, Q498R, N501Y, Y505H, T547K, D614G, H655Y, N679K, P681H, N764K, D796Y, N856K, Q954H, N969K, L981F

Supplementary Figures

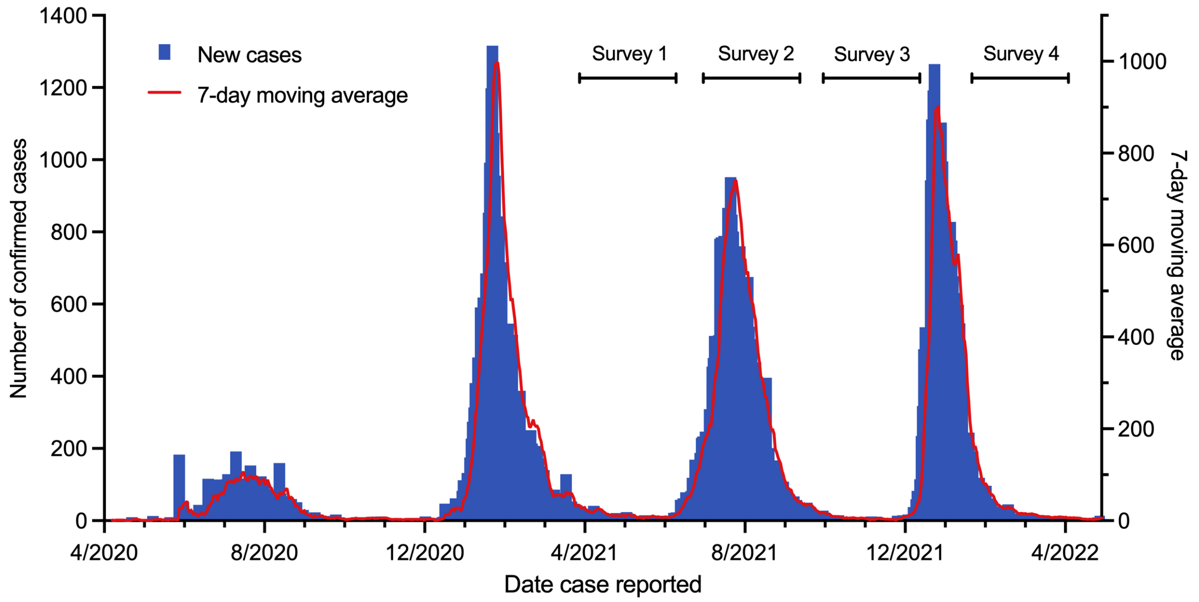

Supplementary Figure 1. Timing of the four survey periods in comparison with the national daily new cases of laboratory–confirmed COVID–19 in Malawi – data from the Public Health Institute of Malawi. The first, smaller peak is thought to be from the Ancestral B.1 virus. Subsequent peaks are due to the Beta, Delta and Omicron BA.1 variants. The amplitude of the B.1 peak versus the subsequent peaks should not be compared due to a lack of routine diagnostic testing at the time of the first wave in Malawi. From Banda et al. (2023), licensed under open access - CC BY 4.9 [10].

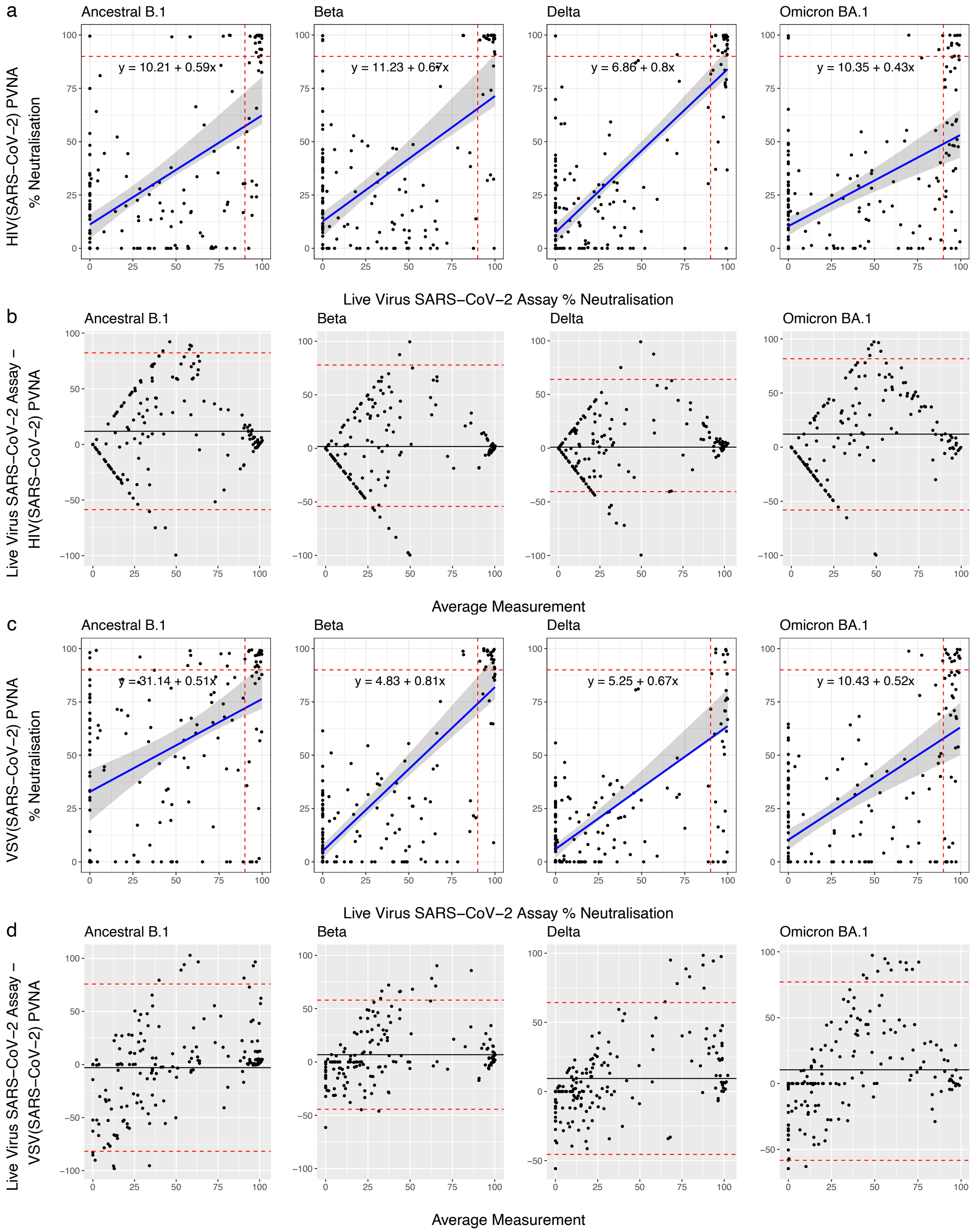

Supplementary Figure 2. Regression analysis and agreement of the HIV(SARS–CoV–2) PVNA and VSV(SARS–CoV–2) PVNA with a live virus SARS–CoV–2 neutralisation assay in HIV-uninfected participants. Sera from HIV–uninfected participants were assessed for neutralising activity against the Ancestral B.1, or variants Beta, Delta, and Omicron BA.1. (A) Linear regression analysis between percent neutralisation generated by the HIV(SARS–CoV–2)–based assay and the live virus SARS–CoV–2 assay for each SARS–CoV–2 lineage. Red dotted lines show the 90% cut–off for both assays. Blue line displays the best fit linear regression line with the grey shaded area showing the 95% confidence interval. Text shows the equation of the regression line in the form y=c+mx where m is

the gradient. (B) Bland–Altman plot showing the agreement between the HIV(SARS–CoV–2)–based assay and the live virus SARS–CoV–2 assay. The black line is the mean difference between the tests for paired samples. The red dotted lines are the 95% limit of agreement. (C) Linear regression analysis between percent neutralisation derived using the VSV(SARS–CoV–2)–based assay and the live virus SARS–CoV–2 assays. Red dotted lines show the 90% cut–off for both assays. Blue line displays the best fit linear regression line with the grey shaded area showing the 95% confidence interval. Text shows the equation of the regression line in the form y=c+mx where m is the gradient. (D) Bland–Altman plot showing the agreement between the VSV(SARS–CoV–2)–based assay and the live virus SARS–CoV–2 assay. The black line is the mean difference between the tests for paired samples. The red dotted lines are the 95% limit of agreement.

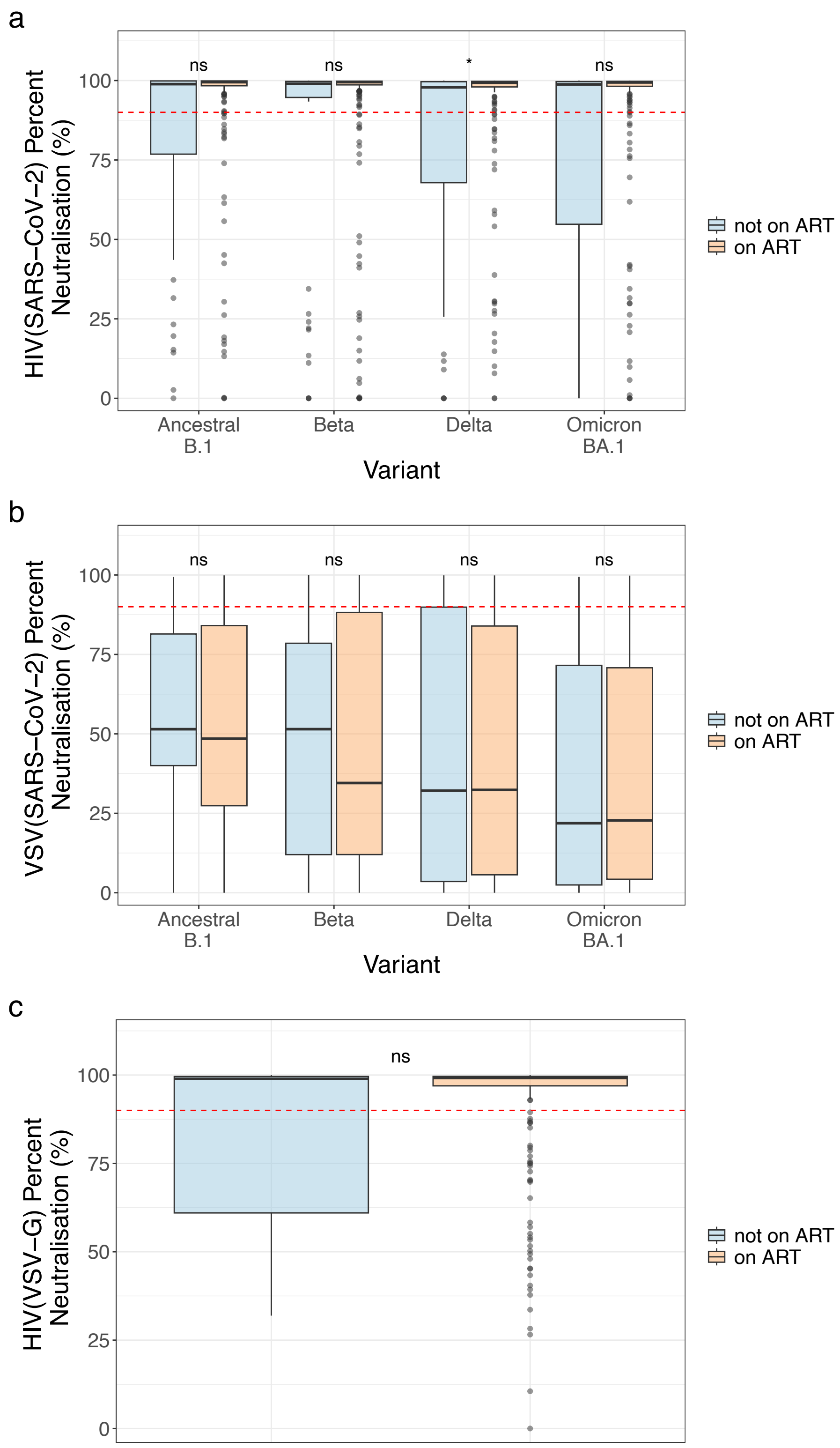

Supplementary Figure 3. Neutralising activity in sera from HIV–infected participants across all four study surveys (Malawi cohort, n=95) using HIV(SARS–CoV–2), VSV(SARS–CoV–2) and HIV(VSV–G) PVNAs, stratified by whether they report receiving antiretroviral therapy (ART) (not on ART – n=17, on ART – n=78). (A) Percent neutralisation using the HIV(SARS–CoV–2) PVNA for Ancestral B.1, Beta, Delta, and Omicron BA.1 variants, stratified by ART treatment (surveys combined). Box plots display the median and interquartile range (IQR). Red dashed line shows the 90% cut–off. Statistical

test used was Wilcoxon rank sum test. (B) Percent neutralisation using the VSV(SARS–CoV–2) PVNA for Ancestral B.1, Beta, Delta, and Omicron BA.1 variants, stratified by ART treatment (surveys combined). Box plots display the median and interquartile range (IQR). Red dashed line shows the 90% cut–off. Statistical test used was Wilcoxon rank sum test. (C) Percent neutralisation using the HIV(VSV–G) PVNA, stratified by ART treatment (surveys combined). Box plots display the median and interquartile range (IQR). Red dashed line shows the 90% cut–off. Statistical test used was Wilcoxon rank sum test. NS – not significant, *p<0·05, **p<0·01, *** p<0·001.

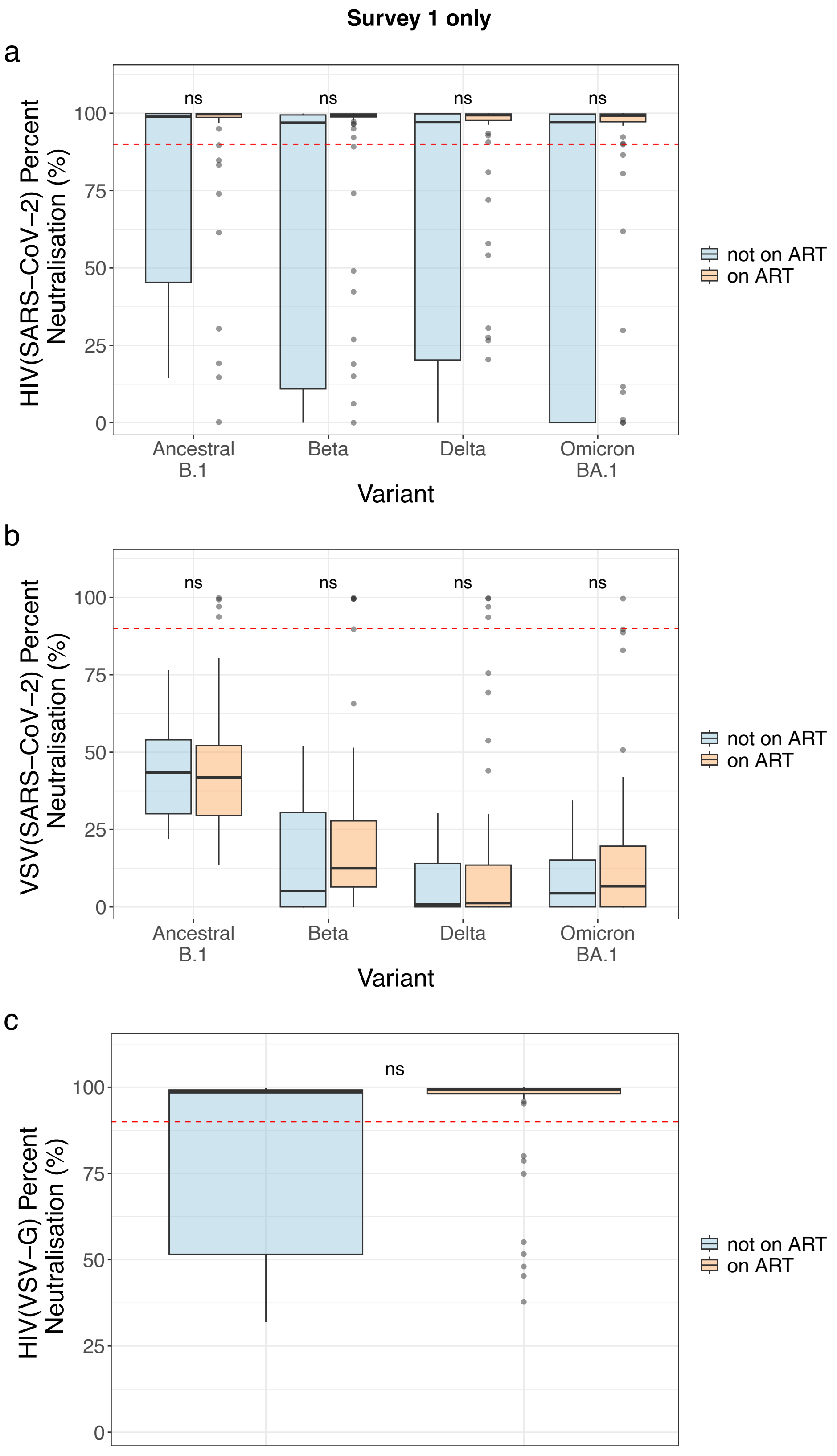

Supplementary Figure 4. Neutralising activity in sera from HIV–infected participants for survey 1 only (Malawi cohort, n=83) using HIV(SARS–CoV–2), VSV(SARS–CoV–2) and HIV(VSV–G) PVNAs, stratified by whether they report receiving antiretroviral therapy (ART) (not on ART – n=13, on ART – n=70). (A) Percent neutralisation using the HIV(SARS–CoV–2) PVNA for Ancestral B.1, Beta,

Delta, and Omicron BA.1 variants, stratified by ART treatment (surveys combined). Box plots display the median and interquartile range (IQR). Red dashed line shows the 90% cut–off. Statistical test used was Wilcoxon rank sum test. (B) Percent neutralisation using the VSV(SARS–CoV–2) PVNA for Ancestral B.1, Beta, Delta, and Omicron BA.1 variants, stratified by ART treatment (surveys combined). Box plots display the median and interquartile range (IQR). Red dashed line shows the 90% cut–off. Statistical test used was Wilcoxon rank sum test. (C) Percent neutralisation using the HIV(VSV–G) PVNA, stratified by ART treatment (surveys combined). Box plots display the median and interquartile range (IQR). Red dashed line shows the 90% cut–off. Statistical test used was Wilcoxon rank sum test. NS – not significant, *p<0·05, **p<0·01, *** p<0·001.

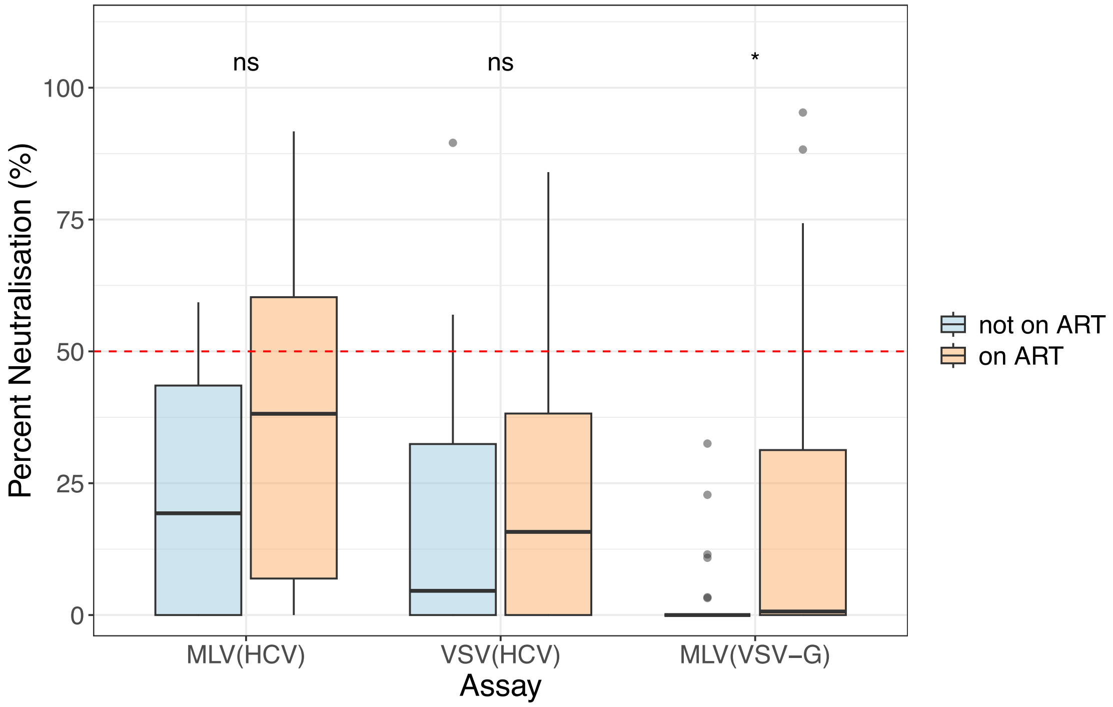

Supplementary Figure 5. Neutralising activity in sera from HIV–infected participants who reported their ART use (UK cohort, n=84) using MLV(HCV), MLV(VSVG) and VSV(HCV) PVNAs, stratified by whether they report receiving antiretroviral therapy (ART) (not on ART – n=25, on ART – n=59). Percent neutralisation using the different assay systems – MLV(HCV), MLV(VSV–G) and VSV(HCV), stratified by ART treatment. Box plots display the median and interquartile range (IQR). Red dashed line shows the 50% cut–off. Statistical test used was Wilcoxon rank sum test: NS – not significant, *p<0·05, **p<0·01, *** p<0·001.

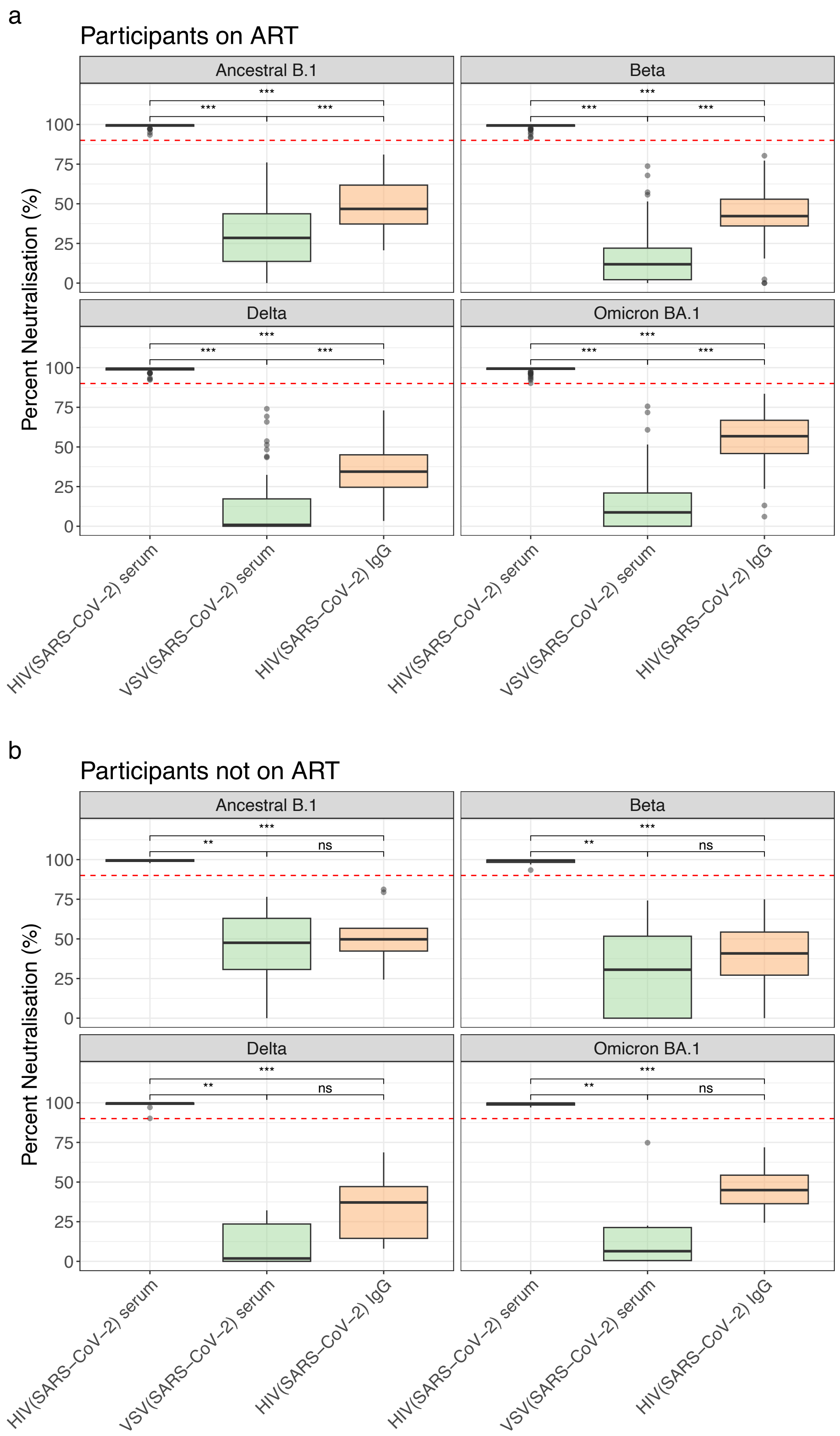

Supplementary Figure 6. Neutralisation of HIV(SARS-CoV-2) and VSV(SARS-CoV-2) by serum and neutralisation of HIV(SARS-CoV-2) by isolated IgG in participants with discrepant neutralisation profiles (Malawi cohort, n=96). (A) Participant on antiretroviral therapy (ART), n=85. (B) Participants

not on ART, n=11. Box plots show percent neutralisation in participants whose serum overestimated HCV neutralisation (i.e., HIV(SARS-CoV-2)-positive but VSV(SARS-CoV-2)-negative). Box plots display the median and interquartile range (IQR). Red dashed line shows the 50% cut–off. Statistical test used was Wilcoxon rank sum test: NS – not significant, *p<0·05, **p<0·01, *** p<0·001.

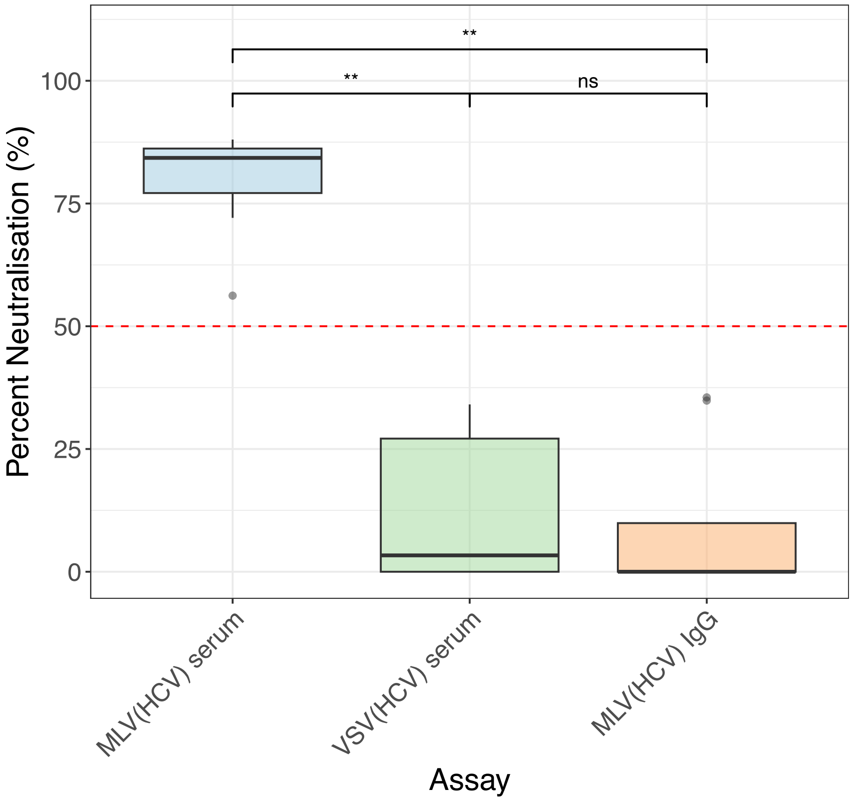

Supplementary Figure 7. Neutralisation of MLV(HCV) and VSV(HCV) by serum and neutralisation of MLV(HCV) by isolated IgG in participants with discrepant neutralisation profiles (UK cohort, n=12). Box plots show percent neutralisation in participants whose serum overestimated HCV neutralisation (i.e., MLV(HCV)-positive but VSV(HCV)-negative). Box plots display the median and interquartile range (IQR). Red dashed line shows the 50% cut–off. Statistical test used was Wilcoxon rank sum test: NS – not significant, *p<0·05, **p<0·01, *** p<0·001.

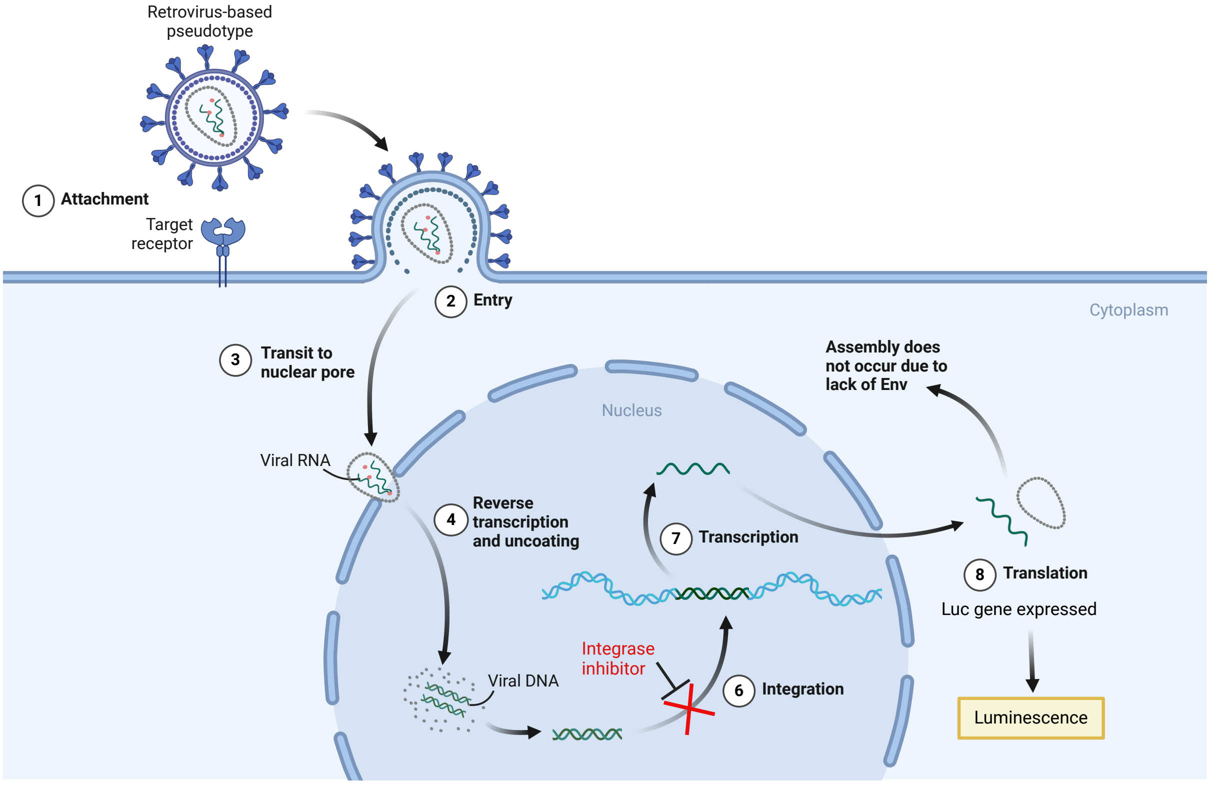

Supplementary Figure 8. Proposed mechanism of interference with the retroviral–based pseudotypes by integrase inhibitors. Integrase inhibitors prevent viral DNA integration and therefore prevent the expression of the luciferase reporter gene. Created with BioRender.com

References for Supplementary Materials

1. Rihn, S. J. et al. A plasmid DNA-launched SARS-CoV-2 reverse genetics system and coronavirus toolkit for COVID-19 research. *PLOS Biol.* **19**, e3001091 (2021).
2. Zufferey, R. et al. Multiply attenuated lentiviral vector achieves efficient gene delivery in vivo. *Nat. Biotechnol.* **15**, 871-875 (1997).
3. Zufferey, R. et al. Self-Inactivating Lentivirus Vector for Safe and Efficient In Vivo Gene Delivery. *J. Virol.* **72**, 9873-9880 (1998).
4. Sušac, L. et al. Structure of a fully assembled tumor-specific T cell receptor ligated by pMHC. *Cell*. **185**, 3201-3213 (2022).
5. Swann, R.E. et al. Broad anti-hepatitis C virus (HCV) antibody responses are associated with improved clinical disease parameters in chronic HCV infection. *J. Virol.* **90**, 4530-4543 (2016).
6. Tarr, A.W. et al. Cloning, expression, and functional analysis of patient-derived hepatitis C virus glycoproteins. In: Sugrue, R.J. (eds) Glycovirology Protocols. Methods in Molecular Biology, vol 379. Humana Press. (2007).
7. Lefrancois, L. et al. The interaction of antibody with the major surface glycoprotein of vesicular stomatitis virus .I. Analysis of neutralizing epitopes with monoclonal antibodies. *Virology.* **121**, 157-167 (1982).

8. Willett, B. J. et al. SARS-CoV-2 Omicron is an immune escape variant with an altered cell entry pathway. *Nat. Microbiol.* **7**, 1161–1179 (2022).
9. Owsianka, A. et al. Monoclonal antibody AP33 defines a broadly neutralizing epitope on the hepatitis C virus E2 envelope glycoprotein. *J. Virol.* **79**, 11095-11104 (2005).
10. Banda, L. et al. Characterizing the evolving SARS-CoV-2 seroprevalence in urban and rural Malawi between February 2021 and April 2022: A population-based cohort study. *Int. J. Infect. Dis.* **137**, 118–125 (2023).
